# Fibrous-layer resident Angptl7^+^ periosteal stem cells sense injury inflammation to orchestrate fracture repair

**DOI:** 10.1038/s41422-025-01202-8

**Published:** 2026-01-08

**Authors:** Bo Jiang, Wenhui Xing, Xiaocui Xu, Shuqin Chen, Heng Feng, Rui Shao, Jiatong Sun, Yazhuo Zhang, Zaiqi Xie, Wenxiang Wang, Xubin Yin, Yi Wang, Miaomiao Wang, Ling Li, Zhong Zhang, Bo Gao, Jinlong Suo, Xuye Hu, Lijun Wang, Jun Sun, Bin Zhou, Bo O. Zhou, Matthew B. Greenblatt, Rongrong Le, Weiguo Zou

**Affiliations:** 1 Key Laboratory of RNA Innovation, Science and Engineering, CAS Center for Excellence in Molecular Cell Science, Shanghai Institute of Biochemistry and Cell Biology, University of Chinese Academy of Sciences, Chinese Academy of Sciences, Shanghai, China; 2 Key Laboratory of Emergency and Trauma of Ministry of Education, Hainan Academy of Medical Sciences, Hainan Medical University, Haikou, Hainan China; 3 Shanghai Key Laboratory of Maternal Fetal Medicine, Clinical and Translational Research Center of Shanghai First Maternity and Infant Hospital, Frontier Science Center for Stem Cell Research, School of Life Sciences and Technology, Tongji University, Shanghai, China; 4 Shanghai Institute of Microsurgery on Extremities, and Department of Orthopedic Surgery, Shanghai Jiao Tong University Affiliated Sixth People’s Hospital, Shanghai, China; 5 Institute of Orthopaedic Surgery, Xijing Hospital, Fourth Military Medical University, Xi’an, Shaanxi China; 6 Key Laboratory of Immune Response and Immunotherapy, School of Basic Medical Sciences, University of Science and Technology of China, Hefei, Anhui China; 7 Key Laboratory of Multi-Cell Systems, CAS Center for Excellence in Molecular Cell Science, Shanghai Institute of Biochemistry and Cell Biology, University of Chinese Academy of Sciences, Chinese Academy of Sciences, Shanghai, China; 8 Department of Pathology and Laboratory Medicine, Weill Cornell Medicine, New York, NY USA; 9 Research Division, Hospital for Special Surgery, New York, NY USA

**Keywords:** Mesenchymal stem cells, Regeneration

## Abstract

Periosteum contains abundant Ctsk-lineage skeletal stem cells (P-SSCs) that are key drivers of intramembranous ossification during bone development and maintenance. However, P-SSCs regenerate fractured bones by mediating endochondral ossification, raising the question of whether distinct P-SSCs subsets separately mediate steady-state bone formation and fracture repair. Here we uncover the heterogeneity of P-SSCs, identifying an *Angptl7*-expressing quiescent P-SSCs subset, which is restricted to the fibrous-layer of periosteum and barely contributes to postnatal bone development. After bone fracture, these cells largely contribute to bone healing by dedicating to endochondral ossification, regenerating the entire bone architecture. Dysfunction of Angptl7-lineage P-SSCs strongly impairs the bone healing process but does not affect steady-state bone formation. Multimodal analysis reveals that these cells can be immediately activated under the regulation of TNF-α/NF-κB signaling, subsequently acquiring osteogenic capacity. Together, our findings unravel an injury-specified P-SSCs subpopulation, providing a model that there are tissue-resident stem cells specialized for injury repair, while parallel stem cells maintain homeostasis.

## Introduction

Bone is one of the few mammalian organs capable of regeneration. Although bone fractures can heal in most cases, there is still a certain probability of non-union occurring.^1,2^ Bone fracture healing is considered to recapitulate the fetal endochondral bone development. In this process, the cartilage primordium is initially formed where skeletal progenitors differentiate into chondrocytes.^3^ The cartilage template is gradually replaced by bony tissue, coordinating with the invasion of perichondrial cells that generate future cortical bone and marrow stromal compartments.^4,5^

Bone development and regeneration both require the involvement of skeletal stem cells (SSCs), which sit at the apex of their differentiation hierarchy and are self-renewing populations giving rise to one or more mature skeletal populations.^6–8^ Previous studies have defined SSCs by cell surface markers.^9,10^ However, emerging evidence suggests that SSCs are heterogeneous in both spatial localization and function, despite expressing similar surface markers.^11,12^ In the growth plate, *Pthrp*-marked SSCs (GP-SSCs) are enriched in the resting zone,^13,14^ which give rise to the proliferating chondrocytes and hypertrophic chondrocytes, contributing to bone growth in puberty.^15–19^ However, bone maintenance in adulthood requires osteogenesis by bone marrow stromal cells, coordinated with *Sstr2*-marked metaphyseal SSCs^20^ and *Fgfr3*-marked endosteal SSCs.^21^

Postnatal periosteum also houses SSCs that contribute to cortical bone formation and injury repair.^22–25^ *Ctsk*, initially being used as a marker for osteoclasts, has recently been recognized for its role in identifying P-SSCs, which form bone directly through intramembranous ossification. Depletion of *osterix* gene (*Osx*, also known as *Sp7*) in Ctsk-lineage P-SSCs impaired periosteal cortical bone formation, with neglectable influence on trabecular bone mass.^22^ Transplantation assays have demonstrated that periosteum is a major cell source for bone fracture repair.^26^ However, P-SSCs mediate fracture healing mainly through endochondral ossification, rather than intramembranous ossification that occurs during homeostasis. Moreover, abnormal activation of Ctsk-lineage P-SSCs can induce both chondrocyte-related metachondromatosis^27^ and osteoblast-related osteosarcoma.^28^ Therefore, it remains unclear whether P-SSCs possess plasticity enabling a bidirectional fate transition, or whether the periosteum houses heterogeneous P-SSCs with some contributing to cortical bone formation via intramembranous ossification and others poised for fracture repair by endochondral ossification.

In this study, we provide evidence that the periosteum houses a unique SSCs lineage, which is quiescent with no steady-state osteogenic ability, but becomes activated in response to inflammatory signals, mediating fracture repair by endochondral ossification. Additionally, we establish novel models for specifically tracing these fracture-repairing key contributors, benefiting future studies to explore the mechanisms underlying fracture non-union.

## Results

### *Angptl7* specifically labels skeletal stem/progenitor cell population 1 (SSPC-1) in the fibrous layer of periosteum

**Fig. 1:**
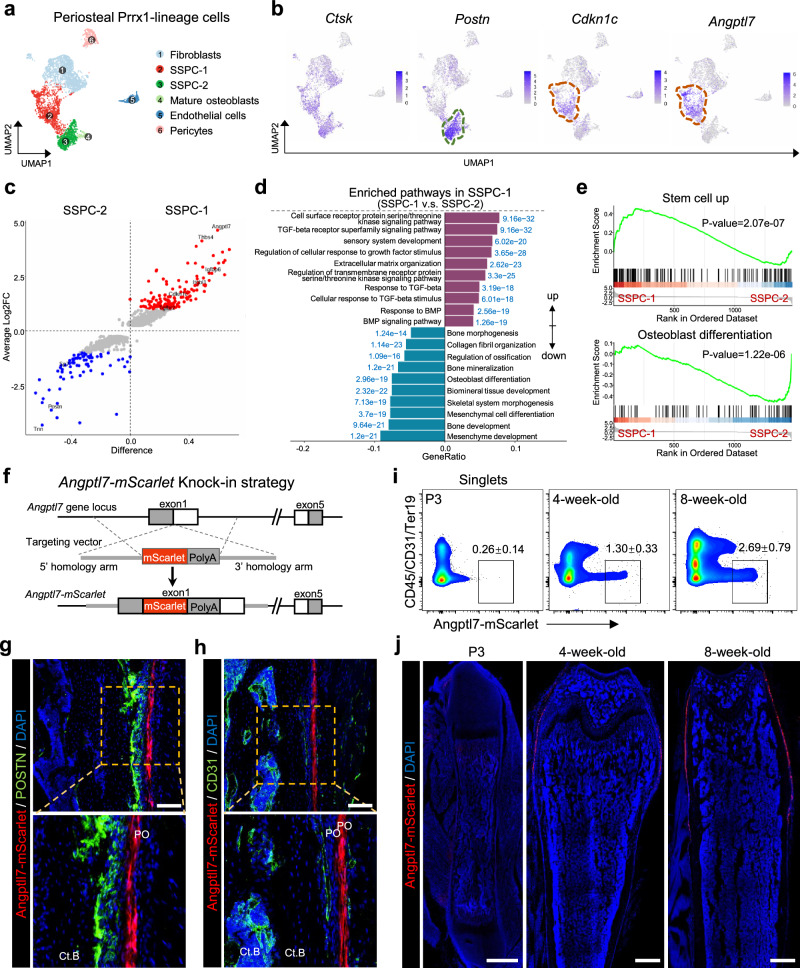
*Angptl7* specifically marks SSPC-1 in the fibrous layer of periosteum. **a** UMAP plots showing the clustering of periosteal Prrx1-Ai9^+^ cells in the scRNA-seq analysis. **b** UMAP plots showing the expression of *Ctsk*, *Postn*, *Cdkn1c*, and *Angptl7*. **c** Mean-Average plots showing the DEGs between SSPC-1 and SSPC-2. Red, highly expressed in SSPC-1; blue, highly expressed in SSPC-2. **d** GO analysis of the DEGs between SSPC-1 and SSPC-2. **e** GSEA analysis showing the enriched gene signature of ‘BOQUEST_STEM_CELL_UP’ in SSPC-1 and ‘GOBP_OSTEOBLAST_DIFFERENTIATION’ in SSPC-2. **f** Schematic diagram of the construction of *Angptl7-mScarlet* mice. **g**, **h** Representative images of femur sections from 8-week-old *Angptl7-mScarlet* mice showing the Angptl7-mScarlet^+^ cells and their relative localization with POSTN (**g**) and CD31 (**h**). Ct.B, cortical bone. PO, periosteum. Scale bar, 100 μm. **i** Representative flow cytometry plots showing the percentage of *Angptl7*-mScarlet^+^ cells in the periosteum of *Angptl7-mScarlet* mice at P3, 4-week-old, and 8-week-old. Data represent mean ± SD. **j** Confocal imaging showing the femur sections from *Angptl7-mScarlet* mice at P3, 4-week-old, and 8-week-old. Scale bar, 500 μm.

To systematically distinguish the cellular composition of the periosteum, we leveraged our existing single-cell RNA sequencing (scRNA-seq) dataset^29^ based on *Prrx1-Cre;Rosa26-Ai9* mice, which mark all the skeletal lineage cells in long bones^30^ and provide robust labelling of periosteum (Supplementary information, Fig. S1a, b). The Prrx1-Ai9^+^ cells were isolated from enzymatically digested periosteum by fluorescence-activated cell sorting (FACS) and subjected to scRNA-seq using the BD Rhapsody™ Single-Cell Analysis system. A total of 4486 cells were retained for analysis after filtering out doublets, low-quality, and blood cells. Non-linear dimensionality reduction with uniform manifold approximation and projection (UMAP) identified 5 clusters as indicated by their key marker genes and differentially expressed genes, including ‘Fibroblasts’ (*Dpt*^+^*Cd34*^+^*Pi16*^+^),^31^ ‘Endothelial cells’ (*Cdh5*^+^*Pecam1*^+^), ‘Pericytes’ (*Acta2*^+^*Myh11*^+^), ‘Mature osteoblasts’ (*Dmp1, Spp1*), a skeletal stem/progenitor cell population defined as ‘SSPC-1’ due to the expression of *Prrx1, Cd200, Pdgfra* and *Ctsk*, as well as another skeletal stem/progenitor cell population defined as ‘SSPC-2’ due to the expression of *Ctsk*, *Postn*, and *Sp7* (Fig. 1a; Supplementary information, Fig. S1c). Specifically, *Ctsk*, as a lineage marker for P-SSCs, was expressed in both SSPC-1 and SSPC-2. *Cdkn1c*, a quiescent stem cell marker,^32^ was highly expressed in SSPC-1, whereas SSPC-2 expressed osteoblast precursor markers, *Postn*^33^ and *Sp7*,^34^ aside from a lower expression of *Cdkn1c* (Fig. 1b; Supplementary information, Fig. S1c). To further elucidate the intrinsic differences in cellular properties between SSPC-1 and SSPC-2, we compared their differential gene expression (DEG) patterns (Fig. 1c–e). We found that SSPC-2 enriched more genes that are associated with ‘Bone development’, ‘Bone mineralization’ and ‘Osteoblast differentiation’ pathways, suggesting its enhanced osteogenic preference compared to SSPC-1. However, SSPC-1-enriched genes were associated with ‘Stem cell up’ and ‘Response to transforming growth factor (TGF)-beta’, which has been considered important for the stem cell maintenance.^35^ We therefore hypothesized that SSPC-2 is the bone-forming P-SSC subset at steady state, but SSPC-1 might represent the resting-state P-SSC subset.

SSPC-1 aroused our great interest, and we sought to identify a genetic marker specific to this population. The ideal marker needs to fulfil two key criteria: 1) distinguish SSPC-1 from SSPC-2; 2) exclude bone marrow-derived SSCs (BM-SSCs) to allow for a precise study of P-SSCs. To address this, we first compared the DEGs between P-SSCs and BM-SSCs, by sorting CD45^–^CD31^–^Ter119^–^CD90^–^6C3^–^CD105^–^CD200^+^ SSCs^9^ from periosteum and bone marrow of wild-type mice for bulk RNA sequencing (RNA-seq). We profiled the genes highly expressed in P-SSCs, with limited expression in BM-SSCs. The heatmap showed the 15 candidate genes (Supplementary information, Fig. S1d–f). We then mapped the candidate genes overlaid on the UMAP plots in the scRNA-seq. We found that *Angptl7* is specifically expressed in the SSPC-1 (Fig. 1b). We also leveraged the published scRNA-seq database of Prrx1-lineage cells from the bone marrow,^21^ in which UMAP plots showed modest expression of *Angptl7* (Supplementary information, Fig. S1g–i). *Angptl7* encodes Angiopoietin-like protein 7, belonging to angiopoietin family, which is usually considered a pro-angiogenic factors. However, other angiopoietin or angiopoietin-like family members show no specific expression in the periosteum or bone marrow (Supplementary information, Fig. S1j, k).

To examine whether *Angptl7* is a suitable target gene for establishing genetic models that precisely trace SSPC-1, we generated an *Angptl7-mScarlet* knock-in reporter allele (Fig. 1f). In adult mice, *Angptl7*-expressing cells were specifically located in the periosteum but were absent in the bone marrow, endosteum, and bone cortex (Fig. 1g). *Angptl7*-expressing cells are also rarely detected in the muscle interstitium, but exist in the tendon (Supplementary information, Fig. S1l, m). We observed that Angptl7-mScarlet^+^ cells exhibit a spindle-shaped morphology, specifically localize to the fibrous layers of periosteum, and are spatially distinct from the Periostin (Postn)-labeling cells in the cambium layer (Fig. 1g). Angptl7-mScarlet^+^ cells also showed no immediate spatial association with CD31^+^ blood vessels (Fig. 1h). Interestingly, the spatial distinction between *Angptl7*-expressing cells and Postn-labeling cells corresponds precisely to the SSPC-1 (*Angptl7*^*+*^) and the SSPC-2 (*Postn*^*+*^) identified in the transcriptomic data.

Moreover, our combined confocal imaging and flow cytometry demonstrated a gradual increase of *Angptl7*-mScarlet^+^ cells during bone development (Fig. 1i, j). *Angptl7*-mScarlet^+^ cells are scarce at P3, but increased in 4-week-old and 8-week-old mice, suggesting that *Angptl7* is predominantly expressed in the late postnatal development stage. Taken together, our results suggested that *Angptl7* can serve as an ideal marker to distinguish fibrous-layer SSPC-1 from cambium-layer SSPC-2, as well as cells from BM sources.

### Angptl7-lineage P-SSCs display minimal contribution to postnatal bone development

**Fig. 2:**
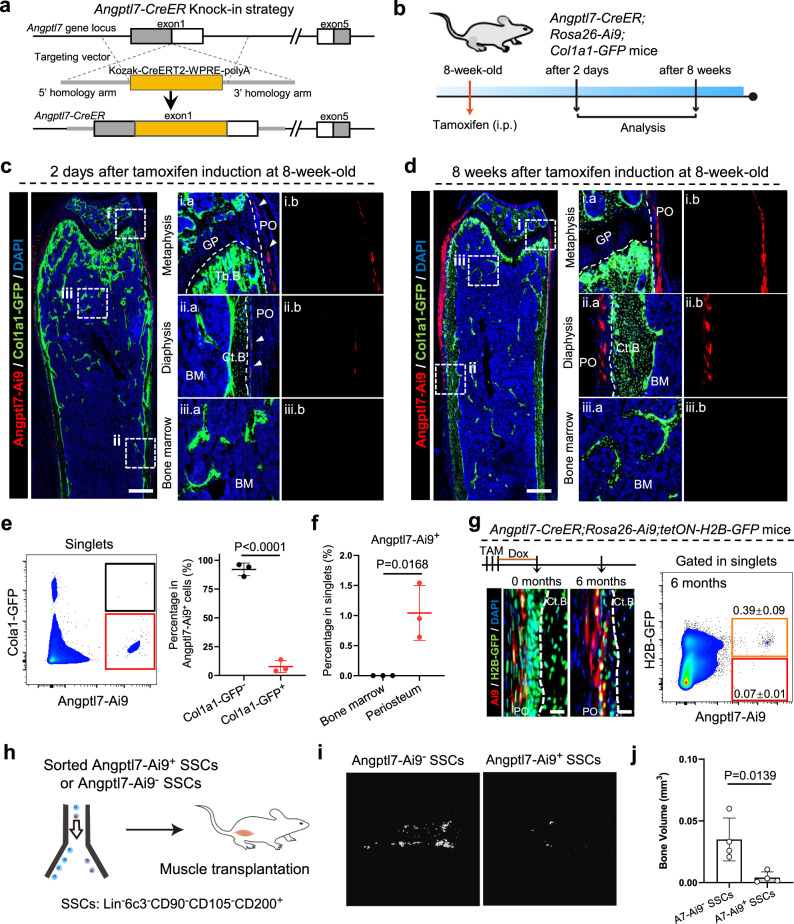
Angptl7-lineage P-SSCs are quiescent and display minimal steady-state osteogenic capacity. **a** Schematic diagram of the construction of *Angptl7-CreER* mice. **b** The diagram of tamoxifen induction strategy. **c**, **d** Confocal imaging of femur sections from *Angptl7-CreER;Rosa26-Ai9;Col1a1-GFP* mice at 2 days (**c**) and 8 weeks (**d**) after tamoxifen treatment at 8-week-old. The indicated region is magnified on the right. GP growth plate, BM bone marrow. White arrowheads indicate the Ai9^+^ cells. Scale bar, 500 μm. **e** Representative FCM plots (left) and quantification (right) of the Col1a1-GFP^+^Angptl7-Ai9^+^ cells and Col1a1-GFP^-^Angptl7-Ai9^+^ in the periosteum from *Angptl7-CreER;Rosa26-Ai9;Col1a1-GFP* mice at 8 weeks after tamoxifen treatment at 8-week-old. Data represent mean ± SD. Two-tailed unpaired *t*-test was used. *n* = 3 mice per condition. **f** Quantification of the Angptl7-Ai9^+^ cells in the periosteum and bone marrow from *Angptl7-CreER;Rosa26-Ai9* mice at 8 weeks after tamoxifen treatment at 8-week-old. Data represent mean ± SD. Two-tailed unpaired *t*-test was used. *n* = 3 mice per condition. **g** Left, representative images of femur sections from *Angptl7-CreER;Rosa26-Ai9;Rosa26-rtTA;TetO-H2B-GFP* mice showing the expression of H2B-GFP in Angptl7-Ai9^+^ cells (label-retaining cells) at 0-month and 6-month after doxycycline treatment. Mice were treated with tamoxifen at 8-week-old, followed by 1-week interval before doxycycline treatment. Scale bar, 10 μm. Right, representative FCM plots and quantification of H2B-GFP^+^Angptl7-Ai9^+^ and H2B-GFP^–^Angptl7-Ai9^+^ cells after a 6-month chase. Data represent mean ± SD. *n* = 3 mice per condition. **h**–**j** Schematic diagram showing the experimental design for transplantation of Angptl7-Ai9^+^ and Angptl7-Ai9^–^CD45^–^CD31^–^Ter119^–^CD90^–^6C3^–^CD105^–^CD200^+^ SSCs (**h**). μCT re-construction (**i**) and quantification (**j**) of the bone organoids at 8 weeks after transplantation are shown. Data represent mean ± SD. Two-tailed unpaired *t*-test was used. *n* = 4 mice per condition.

To systematically fate-map the Angptl7-lineage cells, we next generated an *Angptl7-CreER* genetic mouse model (Fig. 2a), and crossed this with *Rosa26-Ai9* mice and Col1a1*2.3-GFP (hereafter *Col1a1-GFP*, a marker gene for mature osteoblasts) transgenic mice.^36^ Femur sections from 8-week-old *Angptl7-CreER;Rosa26-Ai9;Col1a1-GFP* mice without tamoxifen induction displayed no leaky expression of *Ai9* (Supplementary information, Fig. S2a). We then administered tamoxifen to adult mice at 8 weeks of age (Fig. 2b). After 2 days, echoed with the results from *Angptl7-mScarlet* mice, we observed that Angptl7-lineage cells were specifically located in the periosteum, but not in the growth plate, bone marrow, trabecular bone, cortical bone and endosteum, which were also uniformly Col1a1-GFP^–^ at this time (Fig. 2c). Flow cytometry showed that about 55.73% ± 1.89% CD45^–^CD31^–^Ter119^–^CD90^–^6C3^–^CD105^–^CD200^+^ P-SSCs are marked by Angptl7-Ai9^*+*^ (Supplementary information, Fig. S2b). Immunofluorescence further indicated that Angptl7-lineage cells are present in the fibrous layer of the periosteum, but not co-localized with POSTN, Osterix (OSX) or CD31 (Supplementary information, Fig. S2c–e). After 8 weeks, the Angptl7-Ai9^+^ cells, surprisingly, remained in the periosteum and did not give rise to Col1a1-GFP^+^ osteocytes in the trabecular bone or cortical bone (Fig. 2d). Flow cytometry also confirmed no Angptl7-Ai9^+^ cells in the bone marrow, and rare Angptl7-Ai9^+^Col1a1-GFP^+^ cells in the periosteum at 8 weeks after tamoxifen induction (Fig. 2e, f).

SSCs are conventionally considered crucial for bone maintenance, whereas Angptl7-lineage cells display minimal steady-state osteogenic differentiation, prompting us to further interrogate the stemness of these cells. In vitro colony-forming unit assays demonstrated the robust clonogenic capacity of Angptl7-lineage cells, which also exhibit the ability of tri-lineage differentiation under the differentiation induction medium (Supplementary information, Fig. S2f, g). Furthermore, ‘Tet-on’ (*Rosa26-rtTA;TetO-H2B-GFP*) pulse-chase system revealed preferential label retention of Angptl7-Ai9^+^ cells after a six-month chase period, suggesting a quiescent state of Angptl7-lineage cells (Fig. 2g). These results preliminarily indicate Angptl7-lineage cells fulfil SSCs criteria. We next compared the osteogenic capacity of Angptl7-Ai9^+^ and Angptl7-Ai9^–^CD45^–^CD31^–^Ter119^–^CD90^–^6C3^–^CD105^–^CD200^+^P-SSCs by transplantation assays.^37^ Surprisingly, μCT reconstruction showed that only Angptl7-Ai9^–^ SSCs can form obvious bone architectures, but Angptl7-Ai9^+^ SSCs cannot (Fig. 2h–j). Therefore, our results suggest that *Angptl7* distinguishes a sub-population of P-SSCs with minimal at-steady-state osteogenic ability.

We also fate-mapped Angptl7-lineage cells during postnatal bone development by administering tamoxifen to mice at postnatal day 1 (P1). At P3, Angptl7-lineage cells were observed in the periosteum, but not in the growth plate, trabecular bone, bone marrow, cortical bone or muscle. After 8 weeks, Angptl7-lineage cells were still highly restricted in the periosteum, and rarely gave rise to Col1a1-GFP^+^ osteocytes in the trabecular bone or cortical bone (Supplementary information, Fig. S3a–c). These results were confirmed after tamoxifen induction at 4-weeks of age (Supplementary information, Fig. S3d–f), indicating that Angptl7-lineage cells do not directly participate in early postnatal bone development either. Together, these results support the periosteum-specific residence of Angptl7-lineage P-SSCs, which are quiescent and do not participate in bone formation at steady state.

### Angptl7-lineage P-SSCs choreograph endochondral ossification after fracture

**Fig. 3:**
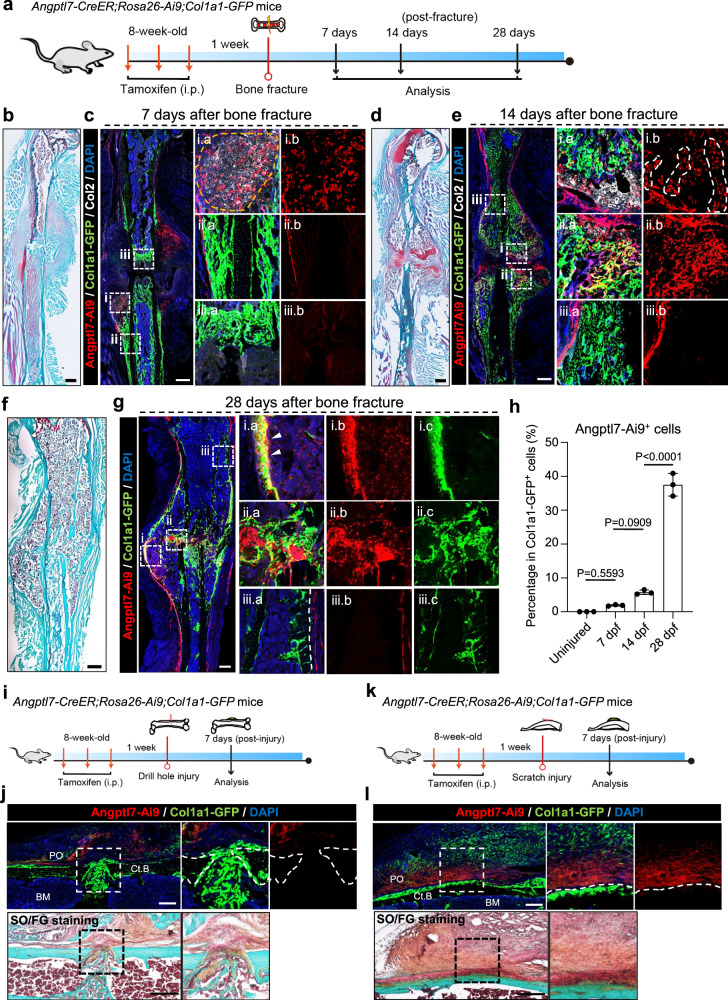
Angptl7*-*lineage P-SSCs largely contribute to bone fracture repair by mediating endochondral ossification. **a** Schematic diagram showing the experimental design for bone fracture assays of *Angptl7-CreER;Rosa26-Ai9;Col1a1-GFP* mice. **b**, **c** Safranin O/fast green (SO/FG) staining (**b**) and confocal imaging (**c**) of femur sections from *Angptl7-CreER;Rosa26-Ai9;Col1a1-GFP* mice at 7 days (7 dpf) after fracture. High-magnification views showing on the right. Dashed line indicated the endochondral ossification region. Scale bar, 500 μm. **d**, **e** SO/FG staining (**d**) and confocal imaging (**e**) of femur sections from *Angptl7-CreER;Rosa26-Ai9;Col1a1-GFP* mice at 14 days (14 dpf) after femur fracture. High-magnification views (right) showing the endochondral ossification region. Scale bar, 500 μm. **f**, **g** SO/FG staining (**f**) and confocal imaging (**g**) of femur sections from *Angptl7-CreER;Rosa26-Ai9;Col1a1-GFP* mice at 28 days after fracture. High-magnification views (right) showing the regenerated region. White arrowheads indicate the regenerated cortex. Scale bar, 500 μm. **h** FCM analysis and quantification of the percentages of Col1a1-GFP^+^ osteoblasts that were Angptl7-Ai9^+^ in uninjured periosteum and in callus at day7, day14 and day28 after fracture. Data represent mean ± SD. one-way ANOVA followed by Tukey’s test. *n* = 3 mice per condition. **i** Schematic diagram of the experimental design for bone drill-hole injury. **j** Confocal imaging (top) and SO/FG staining (bottom) of femur sections from *Angptl7-CreER;Rosa26-Ai9;Col1a1-GFP* mice at day 7 after injury. Scale bar, 250 μm. **k** Schematic diagram of the experimental design for periosteum scratch injury. **l** Confocal imaging (top) and SO/FG staining (bottom) of femur sections from *Angptl7-CreER;Rosa26-Ai9;Col1a1-GFP* mice at day 7 after injury. Scale bar, 250 μm.

Given the limited participation of Angptl7-lineage P-SSCs in steady-state bone formation, we wondered whether these cells mediate bone fracture repair. To this end, we administered tamoxifen to 8-week-old *Angptl7-CreER;Rosa26-Ai9;Col1a1-GFP* mice, followed by middle femoral shaft fracture after a 1-week interval, and analyzed a time course post injury (Fig. 3a). 7 days post fracture (7 dpf), the injury sites were still in the stage of being covered by the cartilaginous callus (Fig. 3b). Angptl7-lineage cells were highly expanded at this time, and contributed to COL2A1^+^ (Col2^+^) chondrocytes formation. However, Col1a1-GFP^+^ osteoblasts/osteocytes were generally not derived from Angptl7-lineage cells at this time (Fig. 3c). 14 days post fracture (14 dpf), Angptl7-lineage cells contributed a lot to the formation of hyaline cartilage intermediate connecting the broken bones (Fig. 3d, e). Interestingly, similar to the columnar chondrocytes in the growth plate, Angptl7-lineage cells in the endochondral ossification region also formed Ai9^+^Col2^+^ columnar chondrocytes, which gradually transformed into Col1a1-GFP^+^ osteoblasts and ossified bones. 28 days post fracture (28 dpf), the cartilaginous callus completely transformed into ossified bone filled with regenerated bone marrow (Fig. 3f). The regenerated bone cortex connecting the fractured bone ends was mostly derived from Angptl7-Ai9^+^ cells, but the bone cortex distant from the fracture site remained Ai9^–^ (Fig. 3g). Flow cytometry analysis of the enzymatically digested uninjured bones or callus from different time points showed the consistent results with immunofluorescence. There were few Angptl7-Ai9^+^Col1a1-GFP^+^ double-positive cells in uninjured bone (0.01% ± 0.00%) and at 7 dpf (1.89% ± 0.17%). The proportion of Angptl7-Ai9^+^Col1a1-GFP^+^ cells gradually increased with the progress of endochondral ossification at 14 dpf (5.78% ± 0.55%) and at 28 dpf (37.53% ± 2.74%) (Fig. 3h).

To further determine whether Angptl7-lineage cells are only involved in endochondral ossification, we also conducted a cortical bone defect and periosteum scratch injury model, which is repaired by intramembranous ossification and endochondral ossification respectively.^38^ In the bone defect model, the Angptl7-lineage cells were highly expanded outside the injury site, but did not give rise to Col1a1-GFP^+^ new bone formation (Fig. 3i, j), suggesting primary contribution from bone marrow stromal cells^39^ or cambium-layer SSPC-2 on this micro-injury. In the periosteum scratch model, Angptl7-lineage cells highly proliferate in the injury site and generate a cartilaginous callus, but these cells rarely contribute to Col1a1-GFP^+^ osteoblasts or fibroblasts (Fig. 3k, l).

Our previous study found that fibrous periosteum mediates endochondral ossification during bone regeneration by using a dual Cre/Dre recombinases-based tracing system (*Pdgfra-CreER;Osx-Dre;ZT1* mice), in which the fibrous layer of the periosteum could be labelled by ZsGreen.^40^ We re-clustered the ZsGreen^+^ cells in the scRNA-seq data of the periosteum from *Pdgfra-CreER;Osx-Dre;ZT1* mice. Interestingly, the ZsGreen^+^ fibrous-layer periosteal cells indeed have heterogeneous cell composition. Among these, *Angptl7* expression was obviously distinguished from the expression of *CD34* which we used to represent the fibrous-layer periosteal cells (Supplementary information, Fig. S4a, b). We next compared the DEGs between Angptl7^+^ cells and CD34^+^ cells (Supplementary information, Fig. S4c). We found that Angptl7^+^ cells enriched genes that are associated with ‘bone development’, ‘cartilage development’, ‘response to transforming growing factor beta pathways’, suggesting that Angptl7^+^ cells exhibit stronger skeletal lineage characteristics compared to CD34^+^ cells. Accordingly, CD34^+^ cell-enriched genes were associated with ‘actin filament organization’ and ‘extracellular structure organization’ (Supplementary information, Fig. S4d, e). We also revisited the scRNA-seq data of periosteal Prrx1-lineage cells, in which *CD34* is highly expressed in fibroblasts instead of SSPCs (Supplementary information, Fig. S4f). Utilizing *CD34-CreER;Rosa26-Ai9* mice, we found that CD34-Ai9^+^ cells are present in the periosteum, bone marrow and muscle at steady-state (Supplementary information, Fig. S4g). Despite the high proliferation of CD34-Ai9^+^ cells in the soft callus at 7 dpf, they modestly gave rise to Col2^+^ chondrocytes and OPN^+^ osteocytes (Supplementary information, Fig. S4h), further suggesting the substantial intrinsic cellular difference between CD34-lineage fibroblasts and Angptl7-lineage P-SSCs.

### Depletion or dysfunction of Angptl7-lineage P-SSCs disrupts fracture repair

**Fig. 4:**
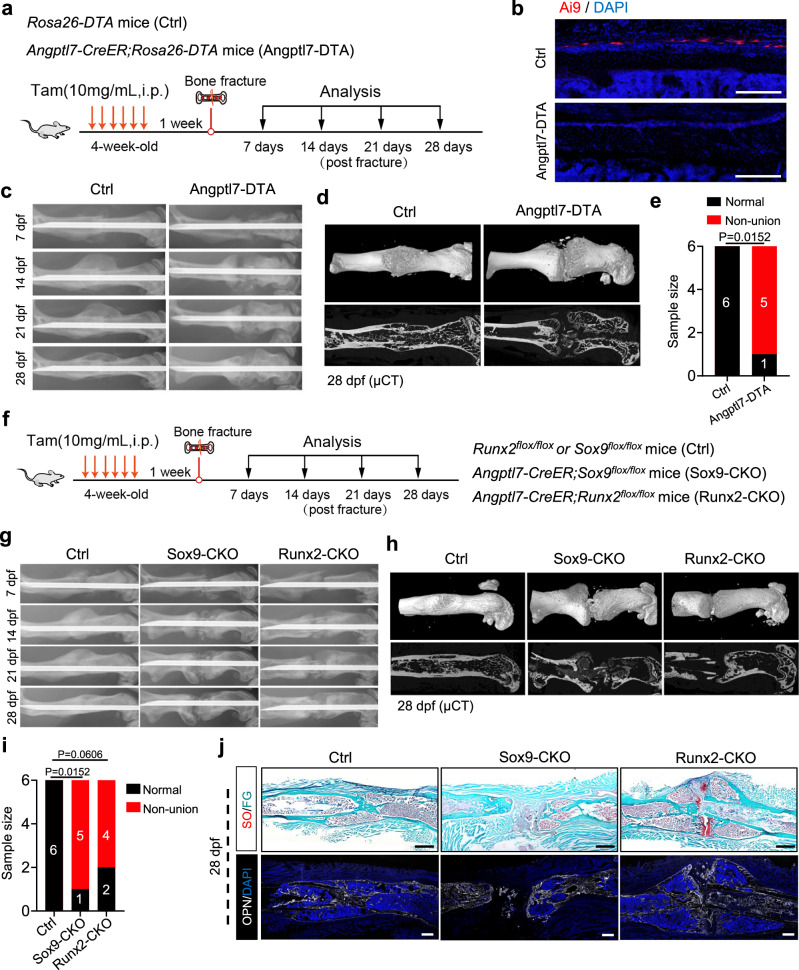
Depletion or dysfunction of Angptl7-lineage P-SSCs disrupts bone fracture repair. **a** Schematic diagram of the experimental design for bone fracture of *Angptl7-CreER;Rosa26-DTA* (Angptl7-DTA) and *Rosa26-DTA* (Ctrl) mice. **b** Confocal imaging of femur sections from *Angptl7-CreER;Rosa26-Ai9;Rosa26-DTA* and *Angptl7-CreER;Rosa26-Ai9* mice showing the depletion of Angptl7-Ai9^+^ cells. Scale bar, 250 μm. **c** Temporal radiographic analysis of the fractured femurs in *Angptl7-CreER;Rosa26-DTA* and *Rosa26-DTA* mice. **d** μCT analysis of the fractured femurs in *Angptl7-CreER;Rosa26-DTA* and *Rosa26-DTA* mice at day28 after fracture. **e** Quantification of the union and non-union femurs after fracture. *n* = 6 mice per condition. Fisher’s exact test was used. **f** Schematic diagram showing the experimental design for bone fracture assays of *Angptl7-CreER;Runx2*^*flox/flox*^ (Runx2-CKO), *Angptl7-CreER;Sox9*^*flox/flox*^ (Sox9-CKO) and *Runx2*^*flox/flox*^ *or Sox9* ^*flox/flox*^ (control). **g** Temporal radiographic analysis of the fractured femurs in Runx2-CKO, Sox9-CKO and control mice. **h** μCT analysis of the fractured femurs from Runx2-CKO, Sox9-CKO and control mice at 28 dpf. **i** Quantification of the union and non-union femurs after fracture. *n* = 6 mice per condition. Fisher’s exact test was used. **j** SO/FG staining (top) and OPN immunostaining (bottom) of femur sections from Runx2-CKO, Sox9-CKO and control mice at 28 dpf. Scale bar, 500 μm.

To verify whether Angptl7-lineage P-SSCs are a major contributor mediating in fracture repair, we crossed *Angptl7-CreER* mice with *Rosa26-DTA* mice,^41^ in which Angptl7-lineage cells could be ablated by expression of the intracellular A subunit of diphtheria toxin after tamoxifen induction (Fig. 4a, b). Phenotypically, X-ray analysis showed impaired fracture healing in *Angptl7-CreER;Rosa26-DTA* mice. In the control group, the hard callus had already begun to form at 14 dpf and transformed into a normal bone cortex at 28 dpf, but the fractured bones did not achieve union in *Angptl7-CreER;Rosa26-DTA* mice (Fig. 4c), which was further illustrated by μCT analysis (Fig. 4d, e). Collectively, these findings indicate that Angptl7-lineage P-SSCs are indispensable for fracture repair by mediating endochondral ossification.

Given the important role of *Sox9* and *Runx2* during endochondral ossification process, we thus examined the functional importance of Angptl7-lineage cells by *Angptl7-CreER;Sox9*^*flox/flox*^ (Sox9-CKO) and *Angptl7-CreER;Runx2*^*flox/flox*^ (Runx2-CKO) conditional knock-out mice (Fig. 4f). Immunostaining confirmed the depletion efficiency of *Sox9* and *Runx2* in Angptl7-lineage cells (Supplementary information, Fig. S5a, b). X-ray imaging indicated impaired bone healing in both Sox9-CKO and Runx2-CKO mice (Fig. 4g). At 28 dpf, in most cases, the fractured bones did not achieve union in Sox9-CKO and Runx2-CKO mice (Fig. 4h, i). Besides, in Sox9-CKO mice, cartilaginous callus formation was impaired, and the fractured bones were still connected by only a fibrous callus at 28 dpf, implying that Angptl7-lineage P-SSCs display a defect in transforming into chondrocytes. However, in Runx2-CKO mice, the fractured bones were connected by cartilaginous tissue and finally lead to the formation of pseudarthrosis, mimicking clinical non-union phenotypes (Fig. 4j). Of note, inconsistent with reduced bone formation in *Ctsk-Cre;Osx*^*flox/flox*^ mice,^22^ dysfunction of Angptl7-lineage cells showed no decreased bone mass without injury, further suggesting that *Angptl7* marks a unique subset of Ctsk-lineage P-SSCs with minimal steady-state osteogenic capacity (Supplementary information, Fig. S5c–i). Together, these results indicated that the involvement and normal function of Angptl7-lineage P-SSCs are essential for bone fracture repair.

### Angptl7-lineage P-SSCs possess the capacity to orchestrate the complete regeneration of bone architecture

**Fig. 5:**
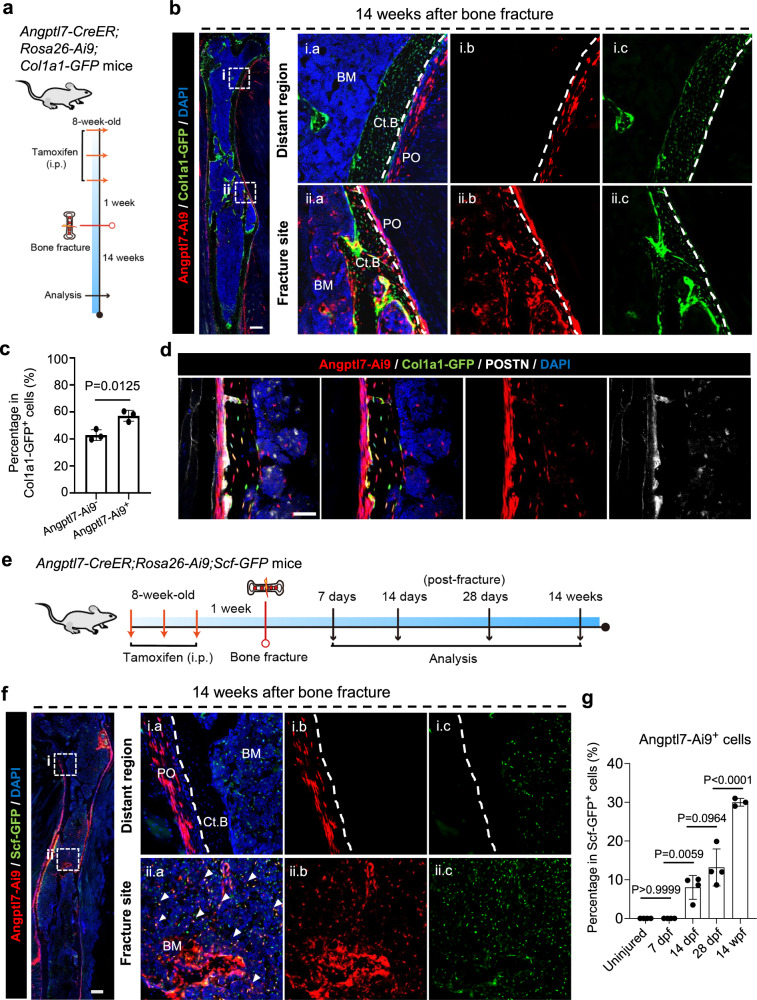
Angptl7-lineage cells can regenerate the entire bone architecture after fracture. **a** Schematic diagram of the experimental design. **b**, **c** Confocal imaging (**b**) and quantification (**c**) of femur sections from *Angptl7-CreER;Rosa26-Ai9;Col1a1-GFP* mice at 14 weeks after femur fracture. High magnification views showing the distant region (i) and adjacent region (ii) from the fracture site. Scale bar, 500 μm. **d** Confocal imaging of femur sections from *Angptl7-CreER;Rosa26-Ai9;Col1a1-GFP* mice at 14 weeks after fracture showing the co-localization of Angptl7-Ai9^+^ cells with POSTN. Scale bar, 50 μm. **e** Schematic diagram of the experimental design. **f** Confocal imaging of femur sections from *Angptl7-CreER;Rosa26-Ai9;Scf-GFP* mice at 14 weeks after fracture. High-magnification views showing the distant region (i) and adjacent region (ii) from the fracture site. White arrowheads indicate the Angptl7-Ai9^+^Scf-GFP^+^ cells. Scale bar, 500 μm. **g** Flow cytometry analysis of uninjured periosteum, callus or healed bone from *Angptl7-CreER;Rosa26-Ai9;Scf-GFP* showing the contribution of Angptl7-lineage cells to Scf-GFP^+^ reticular cells at 7 dpf, 14 dpf, 28 dpf and 14 wpf. Data represent mean ± SD. One-way ANOVA followed by Tukey’s test was used. *n* ≥ 3 mice per condition.

Previous studies about SSCs in fracture repair mainly focus on the early stage, but usually ignore longer-term outcomes. Thus, we fate-mapped the Angptl7-lineage P-SSCs until 14 weeks after fracture (14 wpf), when the injured bones had healed with a relatively normal bone morphology (Fig. 5a). We found that Angptl7-Ai9^+^ cells are major contributors to the regenerated cortical bone. However, the Angptl7-lineage cells in the distant region were still restricted to the periosteum and did not give rise to cortical bone formation (Fig. 5b, c). Notably, Angptl7-lineage cells repopulated both fibrous-layer and cambium-layer periosteum, and contributed to Col1a1-GFP^+^ endosteum in the injured region (Fig. 5b, d).

Moreover, we noticed that Angptl7-lineage cells can invade into bone marrow and repopulate the bone marrow stroma in injured region (Fig. 5b, d). To demonstrate whether Angptl7-lineage P-SSCs contribute to bone marrow regeneration, we crossed *Scf-*GFP reporter mice (Stem cell factor, which is strongly expressed by bone marrow reticular cells and is required for the maintenance of HSPCs^42,43^) with *Angptl7-CreER;Rosa26-Ai9* mice, and followed middle femoral shaft fracture after 1 week interval of tamoxifen treatment (Fig. 5e). 14 weeks after fracture, Angptl7-lineage cells gave rise to Scf-GFP^+^ reticular cells, but these Angptl7-Ai9^+^Scf-GFP^+^ cells mainly restricted in the injury site (Fig. 5f). Flow cytometry showed rare Angptl7-Ai9^+^Scf-GFP^+^ cells in the uninjured bone and in the callus at 7 dpf. Similar to the Angptl7-Ai9^+^Col1a1-GFP^+^ cells, the Angptl7-Ai9^+^Scf-GFP^+^ stromal cells also obviously increased at 14 dpf, and continue increased at 28 dpf and 14 wpf (Fig. 5g). Together, these findings indicate that Angptl7-lineage cells are integral to the comprehensive regeneration of bone architecture following fracture.

### Bone fracture alters the plasticity of Angptl7-lineage P-SSCs

**Fig. 6:**
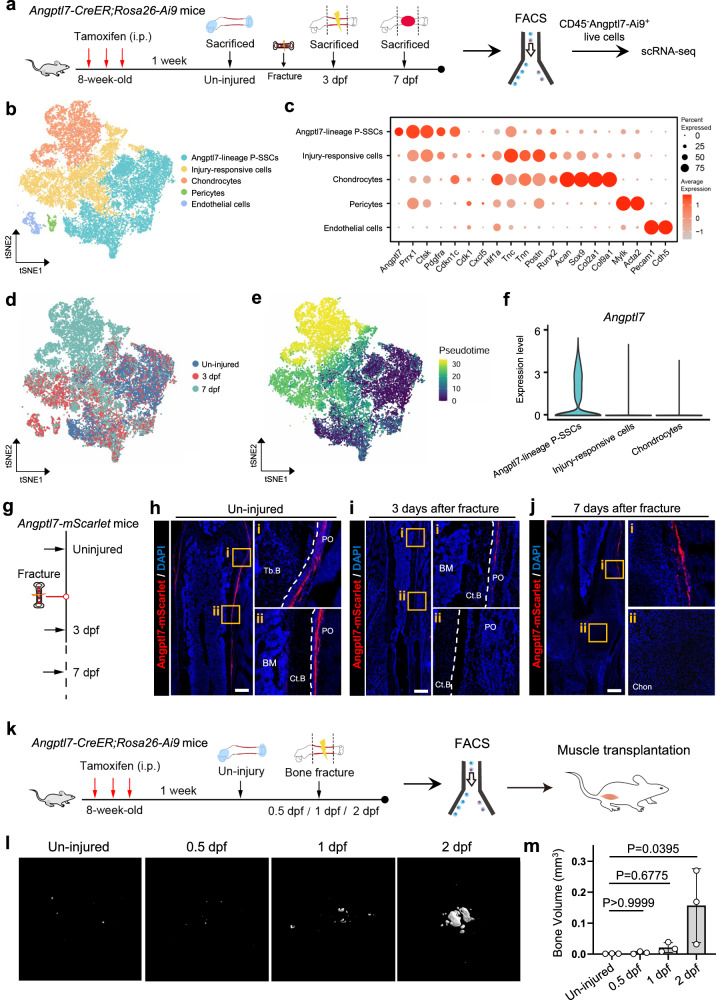
Angptl7-lineage P-SSCs immediately response to bone injury and obtain osteogenic capacity. **a** Schematic diagram of the experimental design for scRNA-seq of Angptl7-lineage cells during bone fracture. **b** *t*-SNE plots visualizing the clustering of total Angptl7-lineage cells from the uninjured femurs, the injured femurs at 3 dpf, and the callus at 7 dpf. **c** Dotplots showing the marker genes for indicated cell populations. **d** *t*-SNE plots colored by samples showing the Angptl7-lineage cells from the uninjured femurs, the injured femurs at 3 dpf, and the callus at 7 dpf. **e** Pseudotime analysis revealing the differentiation trajectories from Angptl7-lineage P-SSCs toward injury-responsive cells and chondrocytes. **f** Violin plots showing the dynamic expression of *Angptl7* across clusters. **g** Schematic diagram showing the experimental design for bone fracture assays of *Angptl7-mScarlet* mice. **h** Confocal imaging of the femur sections showing the Angptl7-mScarlet labelled cells in the uninjured femur. High-magnification views on the right. Scarle bar, 500 μm. **i** Confocal imaging of the femur sections showing the Angptl7-mScarlet labelled cells at 3 dpf. High-magnification views showing the uninjured region (i) and injured region (ii) on the right. Scarle bar, 500 μm. **j** Confocal imaging of the femur sections showing the Angptl7-mScarlet labelled cells at 7 dpf. High-magnification views showing regenerated periosteum (i) and soft callus (ii) on the right. Scarle bar, 500 μm. **k** Schematic diagram of the experimental design for transplantation assays of Angptl7-lineage cells. **l**, **m** μCT re-construction (**l**) and quantification (**m**) of the ossified bone formed by Angptl7-lineage cells from uninjured periosteum and callus at 0.5 dpf/1 dpf/2 dpf at 8 weeks after transplantation. Data represent mean ± SD. Kruskal–Wallis followed by Dunn’s test was used. *n* = 3 mice per condition.

To parallelly demonstrate the plasticity of Angptl7-lineage P-SSCs, we mapped their transcriptional changes before and after injury by scRNA-seq (Fig. 6a). Overall, we captured 29,603 cells that were divided into 5 clusters by *t*-distributed Stochastic Neighbor Embedding (*t*-SNE) plots, in which we identified ‘Chondrocytes’ (*Col2a1*^+^*Acan*^+^*Sox9*^+^), ‘Endothelial cells’ (*Cdh5*^+^*Pecam1*^+^), ‘Pericytes’ (*Mylk*^+^*Acta2*^+^) clusters. ‘Angptl7-lineage P-SSCs’ were identified by their expression of *Angptl7, Prrx1 and Ctsk*. We also identified a population that highly expressed genes related to cell cycle (*Cdk1*), chemokine (*Cxcl5*) and tissue remodeling (*Tnc*, *Tnn*), which were usually up-regulated in activated cells that response to tissue injury,^44^ and we thus defined it as ‘Injury-responsive cells’ (Fig. 6b, c; Supplementary information, Fig. S6a–c). Furthermore, cells clustered by the samples from different timepoints exhibited a consistent gene expression dynamics (Supplementary information, Fig. S6d). Angptl7-lineage P-SSCs were mainly composed of cells from the uninjured group, the injury-responsive cells were mainly composed of cells from 3 dpf and 7 dpf, and the chondrocytes were almost composed of cells from 7 dpf, corresponding with the fate-mapping results that Angptl7-lineage P-SSCs only give rise to endochondral ossification after injury without participation in bone formation at steady state (Fig. 6d; Supplementary information, Fig. S6e). Pseudotime analysis also indicated a trend from Angptl7-lineage P-SSCs to injury-responsive cells and chondrocytes, corresponding to the time course of the samples (Fig. 6e).

Interestingly, we noticed that the expression of *Angptl7* was dramatically decreased upon injury (Fig. 6f). To verify the dynamic expression of *Angptl7* in vivo, we conducted a femoral midshaft fracture on *Angptl7-mScarlet* mice (Fig. 6g). In the uninjured femurs, *Angptl7-mScarlet* labelled cells were specifically located in the periosteum. However, the expression of *Angptl7-mScarlet* near injury sites suddenly disappeared at 3 dpf, and was still absent in the soft callus at 7 dpf (Fig. 6h–j), suggesting that *Angptl7* is immediately down-regulated in activating cells, and only by genetic lineage-tracing models can fate-map these quiescent P-SSCs subset during fracture repair.

The drastically changed transcriptome of Angptl7-lineage cells suggested their altered plasticity after injury. Therefore, we examined the osteogenic activity of Angptl7-lineage cells from the uninjured periosteum or the injured femurs at 0.5 dpf, 1 dpf and 2 dpf by transplantation assays (Fig. 6k). 8 weeks after transplantation, μCT analysis showed that the Angptl7-lineage cells from the uninjured periosteum were unable to form overt bony tissue (Fig. 6l, m), consistent with the results of Angptl7-Ai9^+^ P-SSCs (Fig. 2h–j). However, Angptl7-lineage cells acquired enhanced osteogenic capacity as early as 1 day after injury, and Angptl7-lineage cells from 2 dpf injured femurs formed more overt bony structure (Fig. 6l, m). The simultaneous transcriptional analysis and transplantation assays further support that Angptl7-lineage P-SSCs only undergo osteogenic commitment after a response to injury.

### Inflammatory signals activate Angptl7-lineage P-SSCs after fracture

**Fig. 7:**
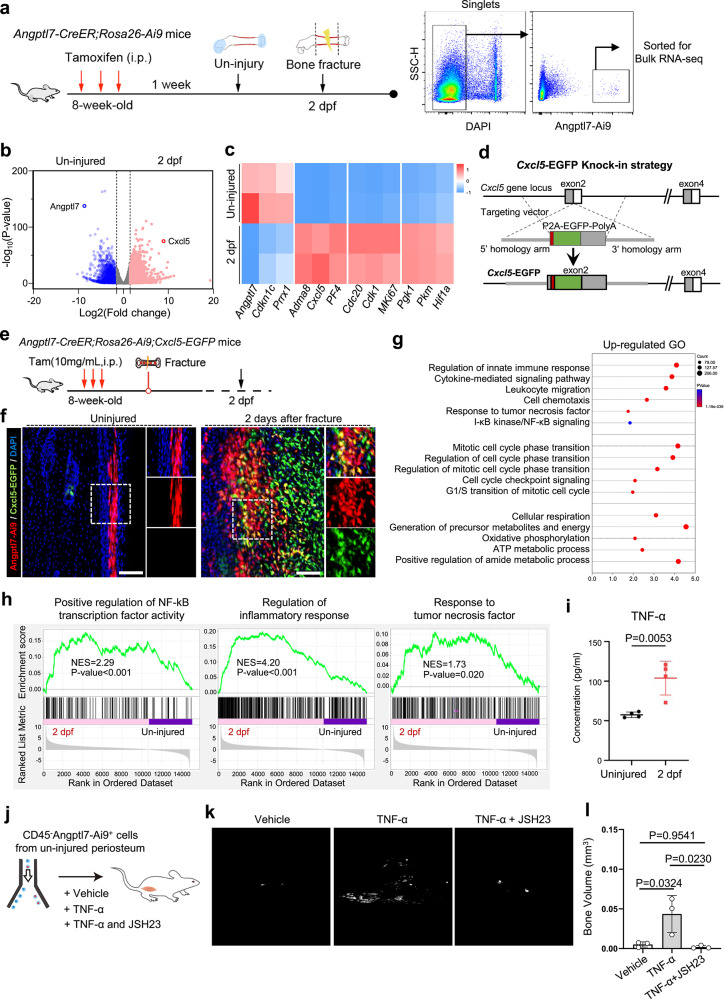
Inflammatory signals trigger the activation of Angptl7-lineage P-SSCs via NF-κB signaling pathway. **a** Schematic diagram of the experimental design for bulk RNA-seq of Angptl7-lineage cells before and after bone fracture. **b** Volcano plots showing the DEGs in the bulk RNA-seq for Angptl7-lineage cells from the uninjured femurs and fractured femurs at 2 dpf. Red, high expressed at 2 dpf; blue, high expressed in uninjured group. **c** Heatmap of the DEGs between Angptl7-lineage cells from the fractured femurs at 2 dpf and the uninjured femurs. Red, high expression; blue, low expression. **d** Construction strategy of *Cxcl5-EGFP* knock-in mice. **e** Schematic diagram of the experimental design for the bone fracture of *Angptl7-CreER;Rosa26-Ai9;Cxcl5-EGFP* mice. **f** Confocal imaging of the femur sections from *Angptl7-CreER;Rosa26-Ai9;Cxcl5-EGFP* mice showing the Angptl7-Ai9^+^ cells and Cxcl5-GFP^+^ cells in the uninjured femur (left) and the injured femur (right) at 2 dpf. Scale bar, 100 μm. **g** GO analysis of the upregulated DEGs in the Angptl7-lineage cells from the injured femurs at 2 dpf. **h** GSEA analysis showing the enriched gene signature in Angptl7-lineage cells from the uninjured femurs and fractured femurs at 2 dpf. **i** Quantification of the concentration of TNF-α in the uninjured femurs and injured femurs at 2 dpf, detected by the electrochemiluminescence assay. Data represent mean ± SD. Two-tailed unpaired *t*-test was used. *n* = 4 mice per condition. **j–l** Schematic diagram of the experimental design (**j**), μCT re-construction (**k**) and quantification (**l**) of the ossified bone formed by Angptl7-lineage cells at 8 weeks after transplantation with vehicle, TNF-α (20 ng/mL) and TNF-α (20 ng/mL) plus JSH23 (10 μM). Data represent mean ± SD. One-way ANOVA followed by Tukey’s test was used. *n* = 3 mice per condition.

Given the rapid response to injury of Angptl7-lineage P-SSCs, we next aimed to explore the mechanism underlying the activation of these cells. We first sorted Angptl7-lineage cells from uninjured periosteum and injured femurs at 2 dpf for RNA-seq (Fig. 7a). Specifically, Angptl7-lineage cells at 2 dpf up-regulated the expression of cell proliferation-related genes (*MKi67, Cdk1, Cdc20*), chemokines (*Cxcl5* and *PF4*) and metabolism-related genes (*Pgk1*, *Pkm*), but down-regulated the *Cdkn1c* and *Angptl7* (Fig. 7b, c). Given the significantly up-regulated expression of *Cxcl5* after fracture, we hypothesized that *Cxcl5* might function as an indicator for the activated Angptl7-lineage cells. We thus generated the *Cxcl5-EGFP* knock-in mice and crossed this with *Angptl7-CreER;Rosa26-Ai9* mice (Fig. 7d). Interestingly, no Angptl7-lineage cells expressed Cxcl5-EGFP in the uninjured femurs. However, at 2 dpf, Angptl7-lineage cells began to express Cxcl5-EGFP, suggesting the injury-specific expression of Cxcl5 (Fig. 7e, f).

Furthermore, GO analysis showed the upregulation of molecular pathways related to immune response, cell cycle and metabolism in Angptl7-lineage cells at 2 dpf (Fig. 7g). We noticed that genes associated with ‘Response to tumor necrosis factor’ and ‘I-κB kinase/NF-κB signaling’ are highly enriched in Angptl7-lineage cells at 2 dpf. Parallelly, GSEA analysis confirmed that Angptl7-lineage cells at 2 dpf enrich the gene signature for the ‘Regulation of inflammatory response’, ‘Positive regulation of NF-κB transcription factor activity’ and ‘Response to tumor necrosis factor’ (Fig. 7h). TNF-α is one of the most potent initiators of inflammatory response, which can activate NF-κB, coordinating tissue regeneration and pro-inflammatory cytokines (including Cxcl5).^45,46^ We thus hypothesized that the inflammatory TNF-α/NF-κB signaling pathway might play a pivotal role in activating Angptl7-lineage P-SSCs. Accordingly, electro-chemiluminescence exhibited a significant increase of TNF-α in the injured callus at 2 dpf (Fig. 7i). We next examined the effects of TNF-α on Angptl7-lineage P-SSCs by transplantation experiments. Surprisingly, TNF-α obviously triggered the osteogenic capacity of Angptl7-lineage cells, as evidenced by pronounced bone formation in TNF-α-treated groups compared to untreated cells. However, the stimulating effect of TNF-α could be removed by NF-κB inhibitor (JSH23) (Fig. 7j–l). Together, these results suggest that TNF-α can trigger the activation of Angptl7-lineage P-SSCs under the regulating of NF-κB signaling.

## Discussion

Here we report a unique sub-population of SSCs in the fibrous layer of periosteum, specifically labelled by Angptl7, which can be distinguished from cambium-layer Postn^+^ SSPC-2 in both spatial and transcriptional levels. Fibrous-layer Angptl7^+^ P-SSCs exhibit attenuated steady-state osteogenic capacity than cambium-layer Postn^+^ SSPC-2. Linage tracing results also revealed that Angptl7-lineage P-SSCs do not participate in postnatal bone formation in homeostasis, but contribute significantly to fracture repair. Moreover, dysfunction of Angptl7-lineage P-SSCs led to serious bone fracture non-union, without obvious bone loss in homeostasis. Therefore, our findings to some extent argue that periosteum houses heterogenous SSCs, including a subset responsible for steady-state bone formation, and another reserved for mediating fracture repair.

Angptl7-lineage P-SSCs are specified to mediate endochondral ossification after bone injury, with rare contribution to intramembranous ossification, as further confirmed by a cortical bone drill hole model and periosteum scratch model. The fibrous-layer specific location of Angptl7-lineage P-SSCs is consistent with our previous finding that the fibrous periosteum mediated endochondral ossification during bone regeneration using *Pdgfra-CreER;Osx-Dre;ZT1* mice. However, due to the broad expression of the *Pdgfra-CreER* in the musculoskeletal system, this mouse model is insufficient to distinguish P-SSCs from fibroblasts and pericytes. Fortunately, the *Angptl7-*based mouse models effectively circumvent this limitation, which specifically marks SSCs in the fibrous layer of periosteum. Therefore, the specific genetic models established here permit faithful lineage tracing for fibrous-layer resident P-SSCs, which will facilitate future research targeting this critical cell population for bone regeneration.

Angptl7 belongs to the angiopoietin family, which is typically associated with angiogenesis. However, scRNA-seq analysis revealed that other angiopoietin or angiopoietin-like members exhibit no specific expression patterns in the periosteum or bone marrow, suggesting a functional divergence between Angptl7 and other angiopoietin family members. Periosteum is derived from embryonic perichondrium, which is pivotal for bone development. Although we did not examine the expression of *Angptl7* during embryonic stages, we observed only scarce Angptl7^+^ cells in the perichondrium at early postnatal age, with their minimal involvement in bone formation. Notably, we observed an increased number of *Angptl7*-expressing cells during postnatal bone development by using the *Angptl7-mScarlet* reporter mice. In other words, the expression of *Angptl7* increases with bone maturation. We thus speculate that a subset of perichondrium-derived progenitors gradually acquires *Angptl7* expression with bone maturation and reside in the fibrous layer of periosteum, where *Angptl7* potentially contributes to their quiescence maintenance. A further implication comes from the rapid down-regulation of cell cycle inhibitory genes in Angptl7-lineage P-SSCs upon injury, such as *Cdkn1c*, which has been reported as a quiescent stem cell marker.^32^ Interestingly, we found that *Angptl7* was also down-regulated after injury, but *Angptl4* was up-regulated (Supplementary information, Fig. S7a), much in the same way as hair follicle stem cells do in skin.^47^ Even though the rapidly decreased expression of *Angptl7*, Angptl7-lineage descendants exhibit a closer spatial association with blood vessels after injury (Supplementary information, Fig. S7b). Therefore, we hypothesize that Angptl7 plays a specific role in preserving SSCs quiescence other than pro-angiogenesis, which might explain why it exhibits sparse expression in osteogenically active cambium layer and bone marrow, but is expressed in the fibrous layer of periosteum. However, direct evidence of Angptl7’s molecular function awaits further detailed validation.

Long-term lineage tracing assays revealed that Angptl7-lineage cells regenerate the neo-cortex that connects the broken bones, and contribute to stromal cells in the bone marrow. We observed that Col1a1-GFP^+^Angptl7-Ai9^+^ osteoblasts were obviously increased with the process of bone healing, as well as the Scf-GFP^+^Angptl7^+^ stromal cells, suggesting the migration of periosteal Angptl7-Ai9^+^ cells into the bone marrow or the trans-differentiation of Angptl7-lineage derived chondrocytes, which implies that the regenerated chondrocytes in callus might undergo trans/dedifferentiation similarly to the chondrocytes in the growth plate.^16,17^ The marrow invasion of Angptl7-lineage cells also appears to recapitulate the invasion of perichondrial cells into the primary ossification center during fetal bone development.

Transcriptional analysis revealed that Angptl7-lineage P-SSCs can respond to the injury signals and transit to an inflammatory-responding state. Inflammatory cytokines, such as TNF-α, can activate Angptl7-lineage P-SSCs, and trigger their osteogenic potential. Therefore, inflammatory signals are critical for the activation of Angptl7-lineage P-SSCs. Recent studies have reported a close relationship between macrophages and stem cells during tissue repair.^48,49^ It will be interesting to investigate whether immune cells could reshape the plasticity of Angptl7-lineage P-SSCs. Due to the dramatic up-regulation of chemokines, it will also be interesting to investigate whether Angptl7-lineage cells themselves can regulate inflammatory immune cells upon injury. Moreover, fracture repair is a complex and finely orchestrated process. Beyond inflammatory signals, it may involve various external cues (e.g. metabolites,^50^ neurotrophic factors^51^) and internal transcription factors that regulate the SSCs’ function. Therefore, future studies may utilize the genetic tools established here to enable further in-depth investigation.

In summary, this study reveals P-SSCs heterogeneity and identifies Angptl7 as a specific marker for fibrous-layer P-SSCs, offering a new target for improving bone fracture repair. The functional dichotomy of Angptl7-lineage P-SSCs before and after fracture provides new insights into the cellular basis for tissue homeostasis maintenance and injury repair.

## Materials and methods

### Mice

*Angptl7-CreER* mice were generated with the assistance of Shanghai Biomodel Organism Co., Ltd. *Angptl7-mScarlet and Cxcl5-EGFP* mice were generated with the assistance of GemPharmatech Co., Ltd. *Prrx1-Cre* mice were purchased from the Jackson Laboratory. *Rosa26-LSL-Ai9* mice were kindly provided by Prof. Zilong Qiu. *Rosa26-DTA* mice were kindly provided by Prof. Yi Arial Zeng. For induction of CreER activity, mice were induced by intraperitoneal injection with 100 μL per 10 g body weight tamoxifen (10 mg/mL, Sigma, T5648).

All mice were maintained under C57BL/6 background in the animal facility at the Center for Excellence in Molecular Cell Science. Sex-matched littermate controls were used in all analyses. Both male and female mice were analyzed. All animals were housed under specific pathogen-free conditions. All mouse procedures were approved by the Institutional Animal Care and Use Committees of the Center for Excellence in Molecular Cell Science, Chinese Academy of Sciences.

### Cell preparation

To prepare periosteal cells from the uninjured femurs, both ends of the femurs were coated with low-melting-point agarose (10% in TAE buffer). Agarose-coated bones were preserved in ice-cold PBS and then digested twice with 1 mg/mL collagenase (MilliporeSigma, C0130), 2 mg/mL dispase II (MilliporeSigma, D4693), and 2% penicillin/streptomycin in minimum essential medium α (α-MEM) (Corning, 10-022-CVR) in a shaker at 37 °C at a speed of 120 rpm for 30 min. To prepare bone cells without the periosteum, periosteum was first carefully scraped away using a disposable scalpel. After cutting both ends of the femurs, the bone shaft was cut into pieces by scissors and then digested twice at 37 °C for 30 min, as above. To prepare cells in the injured femurs, the callus was cut into pieces by scissors and then digested twice at 37 °C for 30 min, as above. After digestion, the cell suspension was filtered through a 40-µm nylon mesh (BD Falcon, BD Biosciences, 352340), incubated with RBC lysis buffer (Beyotime, C3702) and washed twice in ice-old PBS.

### Flow cytometry

For flow cytometric analysis, Angptl7-Ai9^+^ cells from *Angptl7-CreER;Rosa26-Ai9* mice, were equally added into each individual tube containing different antibodies for immunostaining using a previously described method.^52^ Freshly prepared cells were stained with PerCP/Cy5.5-conjugated anti-CD31 (Biolegend, 102420), APC-conjugated anti-CD45 (Biolegend, 103112), PerCP/Cy5.5-conjugated anti-CD45 (Biolegend, 103132), PerCP/Cy5.5-conjugated anti-TER-119 (Biolegend, 116228), FITC-conjugated anti-6C3/Ly-51 (Biolegend, 108305), Brilliant Violet 605-conjugated anti-CD90.2 (Biolegend, 140318), PE/Cy7-conjugated anti-CD105 (Biolegend, 120410), APC-conjugated anti-mouse CD200 (Biolegend, 123810). After washes with PBS, flow cytometry was conducted by CytoFlex LX (Beckman), and the data were analyzed by FlowJo software (v10.8.1). Cell sorting was performed on AriaFusion (BD Biosciences) and MA900 (SONY).

### scRNA-seq and analysis

scRNA-seq was performed with help from NovelBio Bio-Pharm Technology Co., Ltd. A BD Rhapsody™ Single-Cell Analysis system was used to capture the transcriptomic information of the sorted cells. Single-cell capture was achieved by random distribution of a single-cell suspension across > 200,000 microwells through a limited dilution approach. Beads with oligonucleotide barcodes were added to saturation so that a bead was paired with a cell in a microwell. The cells were lysed in the microwell to hybridize mRNA molecules to barcoded capture oligos on the beads. Beads were collected into a single tube for reverse transcription and ExoI digestion. Upon cDNA synthesis, each cDNA molecule was tagged on the 5′ end (that is, the 3′ end of the mRNA transcript) with a unique molecular identifier and cell barcode indicating its cell of origin. Whole transcriptome libraries were prepared using the BD Rhapsody single-cell whole-transcriptome amplification (WTA) workflow including random priming and extension (RPE), RPE amplification PCR and WTA index PCR. The libraries were quantified using a High Sensitivity DNA chip (Agilent) on a Bioanalyzer 2200 and the Qubit High Sensitivity DNA assay (Thermo Fisher Scientific). Sequencing was performed with an Illumina sequencer (Illumina, San Diego, CA) on a 150 bp paired-end run.

We applied FASTQ files with default parameter filtering the adaptor sequence and removed the low-quality reads to achieve clean data. After pre-processing, expression matrices were loaded into R (v4.1.2) with Seurat (v4.3.0). We concatenated the count matrices from all the samples and only kept good quality cells that met the following criteria: 1) cells with between 1000 and 5000 genes expressed; 2) cells with mitochondrial gene expression percentages of fewer than 25. Genes expressed in fewer than 10 cells were removed. Overall, 29,603 single cells with a mean 2119 genes per cell were retained. Integration of 10X Genomics datasets was performed following Seurat’s integration pipeline. Specifically, each 10X Genomics library was one batch. Datasets were scaled and log-transformed using ‘NormalizeData()’ function. For integration, the 2000 most-variable genes were identified by ‘FindVariableFeatures()’ with parameters: selection.method = “vst”, nfeatures = 2000. Integration anchors were identified based on these genes using canonical correlation analysis (CCA) integration tool with 30 dimensions as implemented in the ‘FindIntegrationAnchors()’ function. The data were then integrated using ‘IntegrateData()’ and scaled again using ‘ScaleData()’. Principal component analysis (PCA) with 30 principal components was performed by ‘RunPCA()’, and *t*-SNE dimension reduction with 20 principal components was performed by ‘RunTSNE’. A nearest-neighbor graph using the 20 dimensions of the PCA reduction was calculated using ‘FindNeighbors()’, followed by clustering using ‘FindClusters()’ with a resolution of 0.4. Seurat’s ‘DimPlot()’ was used to plot cell clusters and ‘FeaturePlot()’ function was used to demonstrate individual gene expression on UMAP embedding. Seurat’s ‘DotPlot()’ was used to plot average expression for marker genes. The clusters were identified as different major cell lineages based on the average gene expression of well-known markers.

### Bulk RNA-seq

Bulk RNA-seq were conducted using the previously described method.^52^ Briefly, Angptl7-lineage cells were directly sorted into TRIzol ls buffer (Invitrogen, 10296-028), about 2 ×10^4^ cells were collected for each sample. RNA-seq library preparation were conducted according VAHTS® Universal V6 RNA-seq Library Prep kit manual (Vazyme, NR605-01). The size of the libraries was selected by using the DNA Clean beads (Vazyme, N411-01), with average size of 300 bp. The libraries were sequenced using Illumina HiSeq 4000 (pair end 50 bp). The RNA sequencing reads were aligned to GRCm38.98 Mus musculus reference genome with STAR (v2.7.2a) using a supplied set of known transcripts in GTF format (RefSeq GRCm38.98; Mus musculus, Ensembl). Differentially expressed genes were calculated using fold change with cutoff (Log Fold change greater than 1.5, and RPKM greater than the lower quartile), and RPKM values were calculated with a custom R script.

### Immunostaining and confocal imaging

For immunofluorescence staining, frozen sections were air-dried, rehydrated with PBS, and then blocked and permeabilized with 3% BSA and 0.2% Triton X-100 in PBS at room temperature for 1 h. Sections were probed with the following primary antibodies overnight at 4 °C: rabbit anti-Periostin (R&D, AF2955, 1:200), goat anti-Osteopontin (R&D, AF808, 1:500), mouse anti-Col2a1 (Abcam, ab185430, 1:200; Boster, BA0533, 1:200), anti-Osx (Abcam, ab209484, 1:100), anti-CD31 (R&D, AF3628, 1:200), anti-Sox9 (Millipore, AB5535, 1:100), and anti-Runx2 (Santa Cruz, sc-390351, 1:100). Fluorescent dye-labeled secondary antibodies, including donkey anti-rabbit Alexa Fluor 647 (Molecular Probes, A21206, 1:1000), donkey anti-goat Alexa Fluor 647 (Molecular Probes, A11055, 1:1000), and donkey anti-mouse Alexa Fluor 647 (Molecular Probes, A21202, 1:1000), were incubated with sections for 1 h at room temperature after washing. Nuclei were counterstained with DAPI (Sigma, D9542). Sections were mounted using fluorescence mounting medium (Dako, S3023).

### In vitro analysis of multipotent differentiation

For osteogenic differentiation, FACS sorted Angptl7-Ai9^+^ cells were cultured in osteoblast induction medium (α-MEM containing 10% FBS, 5 mM β-glycerophosphate (Sigma, G9422), 50 µg/mL L-ascorbic acid (Sigma, A5960), and 1% penicillin/streptomycin (Gibco, 15140-122)). The medium was changed every three days. After seven days of induction, cells were fixed with 10% neutral buffered formalin (Sigma, HT501320) and stained with the BCIP/NBT ALP staining kit (Beyotime, C3206) according to the manufacturer’s instructions.

For chondrogenic differentiation, a micromass culture method was used to determine the chondrogenic differentiation ability. A total of 5 × 10^6^ sorted cells per mL were plated as a 10-μL micromass drop in a culture well of a 24-well plate and incubated with 5% CO2 at 37 °C for 2 h to allow cell attachment. Then, 1 mL of chondrocyte induction medium (α-MEM containing 10% FBS, 1% ITS (Cyagen, ITSS-10201-10), 10 ng/mL TGFβ3 (Peprotech, 100-36E), 100 nM dexamethasone (Sigma, D1756), 1 mM sodium pyruvate (Sigma, 25-000-CIR), 40 μg/mL proline (Sigma, P5607), 50 μg/mL L-ascorbic acid 2-phosphate (Sigma, A8960), and 1% penicillin/streptomycin) was added and incubated for four days. The micromass was acidified with 0.1 N HCl and stained with 1% Alcian blue (Sigma, A5268).

For adipogenic differentiation assays, FACS sorted Angptl7-Ai9^+^ cells were cultured in the nutrient medium with adipogenic differentiation medium (Cyagen, MUBMX-90031) for another 12 days. Adipocyte formation was detected by staining with Oil red O (Sangon Biotech, A600395-0050).

### Safranin O/fast green staining

Femur sections were sequentially immersed in the following solutions: hematoxylin solution for 5 min, double-distilled water for 5 min, 0.2% fast green solution (Sigma-Aldrich, F7252) for 3 min, 1% acetic acid (Sangon, A501931) in water for 10 s and 0.1% safranin O solution (Sigma-Aldrich, S2255) for 2 min. The slides were then dehydrated, cleared, and mounted in neutral mounting medium (Sigma, 317616). Images were acquired on a BX53 microscope (Olympus).

### Muscle transplantation model

Intramuscular transplantation was performed in 8-week-old nude mice hosts for transplantation of cells of interest. Sorted cells were resuspended with growth reduced-matrigel (Corning, 354230) after centrifugation. 1 ×10^4^ of the indicated cells were transplanted. For the treatment of TNF-α or NF-κB inhibitor, 20 ng/mL recombinant TNF-α protein (Abclonal, RP01071) or 10 μM JSH-23 (MCE, HY-13982) was added into the cell suspension. A 1-mm longitudinal incision was made on the hindlimb, and the right anterolateral femur was exposed. The vastus lateralis muscle was dissected and a 2–3 mm muscle pouch was surgically created. A surgifoam absorbable gelatin sponge (Ethicon, 1972) containing cells in Matrigel was placed into the muscle pouch. The muscles were repositioned, and the skin was closed using a 6/0 nylon suture. Mice were sacrificed after 8 weeks post-surgery.

### Mouse femoral bone fracture model and X-ray analysis

Mice were anesthetized using chloral hydrate. The patella was dislocated laterally to expose the femoral condyles. An intramedullary pin (25 G needle) was inserted into intramedullary canal of the femur to stabilize the impending fracture, and the patella was then relocated. The fracture was generated using a dentist’s microdrill at the midpoint of the femur. The muscles were repositioned, and the skin was closed using a 6/0 nylon suture. Fracture repair was followed radiographically using an Faxitron® MX-20 Cabinet X-ray System.

### Bone drill hole and periosteum scratch model

Bone drill hole model was established with a drill-hole injury of cortical bone in the middle of the antero-medial tibial. A skin incision was made at the middle of tibia. Blunt dissection of the subcutaneous tissue was performed until the periosteum was exposed. A needle that was 0.7 mm in diameter was used to drill a hole into the anterior cortices. Afterward, the subcutaneous tissue was repositioned, the skin was closed using a 6/0 nylon suture.

Periosteum scratch model was established with a scratch of the periosteum in the middle of the antero-medial tibial. After the mice were anesthetized and the periosteum was exposed, a needle tip was used to scratch the periosteum lengthwise in the middle of the left anteromedial tibia. The length of the scratch was 0.5–1 cm. Afterwards, the subcutaneous tissue was repositioned, and skin was closed using a 6/0 nylon suture.

### Cytokine measurement

Concentration of TNF-α from un-injured femurs and fractured femurs at 2 dpf was analyzed with the Meso Scale Discovery V-PLEX Proinflammatory Panel1 (mouse) Kit (K15048D; Meso Scale Discovery) according to the manufacturer’s instructions.

### μCT analysis

Femurs isolated from age- and sex-matched mice were fixed with 70% ethanol and scanned using a SkyScan1272 at a 9-μm resolution for quantitative analysis. Three-dimensional images were reconstructed using a fixed threshold.

### Quantification and statistical analysis

Statistical analysis was performed using GraphPad Prism 9 software (GraphPad Software). The data are presented as the means ± SD. Two groups were compared using two-tailed Student’s *t*-tests. Ordinary one-way ANOVA test was used to compare more than two groups unless otherwise stated. *P* < 0.05 was considered to be a statistically significant difference.

## Supplementary information

Supplementary information, Fig.S1. Identification of *Angptl7* as a specific marker for periosteal SSPC-1

Supplementary information, Fig.S2. Identification of Angptl7-lineage cells as P-SSCs in the fibrous-layer of periosteum

Supplementary information, Fig.S3. Angptl7-lineage P-SSCs display minimal participation in postnatal bone development

Supplementary information, Fig.S4. Angptl7-lineage P-SSCs are basically distinguished from CD34-lineage fibroblasts

Supplementary information, Fig.S5. Functional perturbation of Angptl7-lineage P-SSCs exhibits negligible effects on bone homeostasis

Supplementary information, Fig.S6. ScRNA-seq analysis of the Angptl7-lineage cells during fracture repair

Supplementary information, Fig.S7. Dynamic expression of angiogenic factors in Angptl7-lineage cells during fracture repair

## Data Availability

scRNA-seq data and bulk RNA-seq data are deposited at GEO/GSA with accession numbers: GSE249206 (GEO), CRA020553 (GSA).
