# Supplementary material for: Fibrous-layer resident Angptl7+ periosteal stem cells sense injury inflammation to orchestrate fracture repair
Source: Cell Res. 2026 Jan 8;36(2):121–36. doi: 10.1038/s41422-025-01202-8 (PMC12847966; doi:10.1038/s41422-025-01202-8)
Supplement: Supplementary file 1 — Supplementary information, Fig.S1. Identification of Angptl7 as a specific marker for periosteal SSPC-1 [file 41422_2025_1202_MOESM1_ESM.pdf]

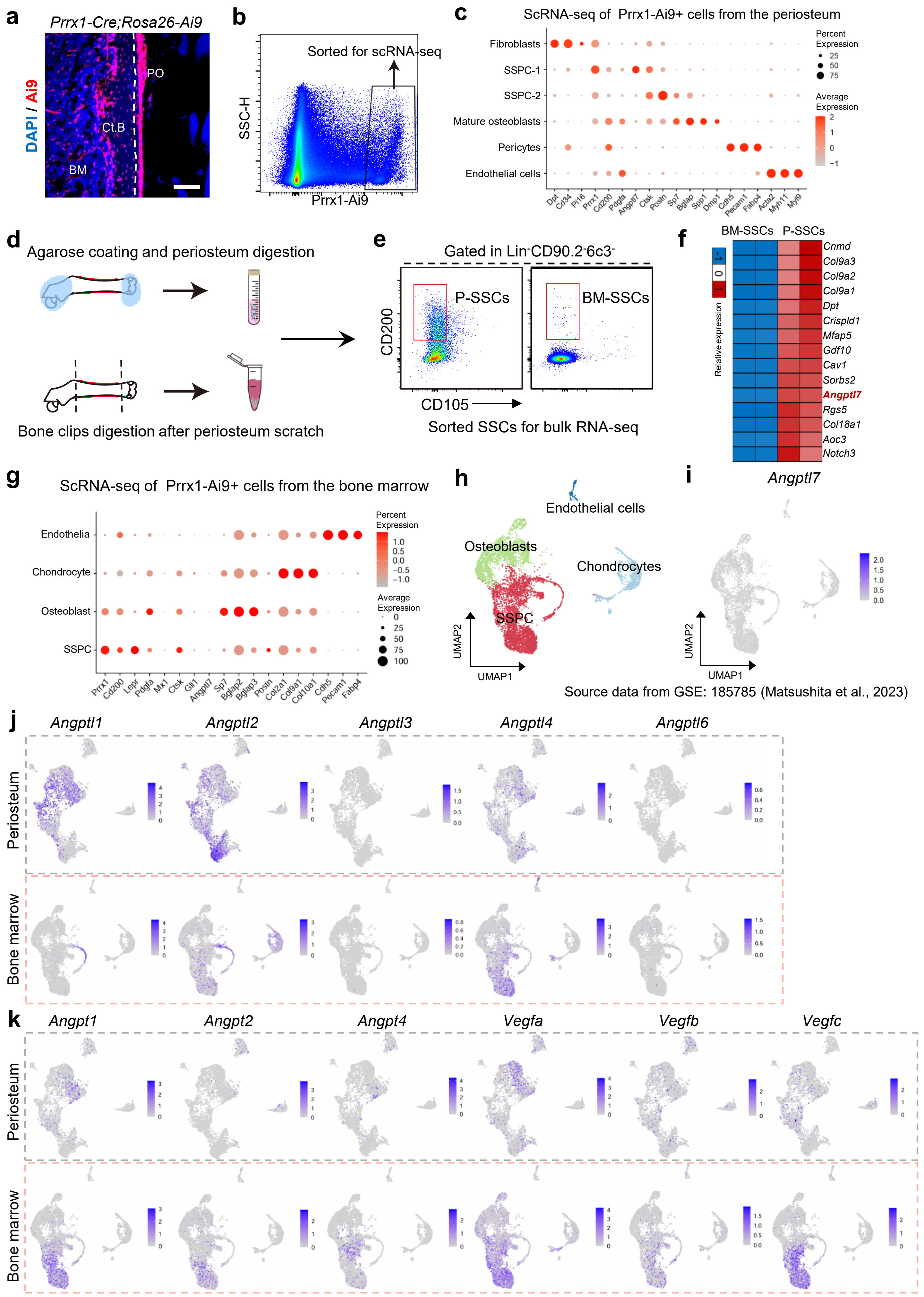
a
Prrx1-Cre;Rosa26-Ai9
DAPI / Ai9
PO
Ct.B
BM
b
Sorted for scRNA-seq
SSC-H
Prrx1-Ai9
c
ScRNA-seq of Prrx1-Ai9+ cells from the periosteum
Fibroblasts
SSPC-1
SSPC-2
Mature osteoblasts
Pericytes
Endothelial cells
Dpt Cd34 Pi16 Prrx1 Cd200 Pdgfa Angptl7 Ctsk Postn Sp7 Bglap Spp1 Dmp1 Cdh5 Pecam1 Fabp4 Acta2 Myh11 Myl9
Percent Expression
25
50
75
Average Expression
2
1
0
−1
d
Agarose coating and periosteum digestion
Bone clips digestion after periosteum scratch
e
Gated in Lin-CD90.2-6c3-
P-SSCs
BM-SSCs
CD200
CD105
Sorted SSCs for bulk RNA-seq
f
BM-SSCs
P-SSCs
-1
0
1
Relative expression
Cnmd
Col9a3
Col9a2
Col9a1
Dpt
Crispld1
Mfap5
Gdf10
Cav1
Sorbs2
Angptl7
Rgs5
Col18a1
Aoc3
Notch3
g
ScRNA-seq of Prrx1-Ai9+ cells from the bone marrow
Endothelia
Chondrocyte
Osteoblast
SSPC
Prrx1 Cd200 Lepr Pdgfa Mx1 Ctsk Gli1 Angptl7 Sp7 Bglap2 Bglap3 Postn Col2a1 Col9a1 Col10a1 Cdh5 Pecam1 Fabp4
Percent Expression
1.0
0.5
0.0
−0.5
−1.0
Average Expression
0
25
50
75
100
h
Endothelial cells
Osteoblasts
Chondrocytes
SSPC
UMAP2
UMAP1
i
Angptl7
2.0
1.5
1.0
0.5
0.0
UMAP2
UMAP1
Source data from GSE: 185785 (Matsushita et al., 2023)
j
Angptl1
Angptl2
Angptl3
Angptl4
Angptl6
Periosteum
Bone marrow
k
Angpt1
Angpt2
Angpt4
Vegfa
Vegfb
Vegfc
Periosteum
Bone marrow

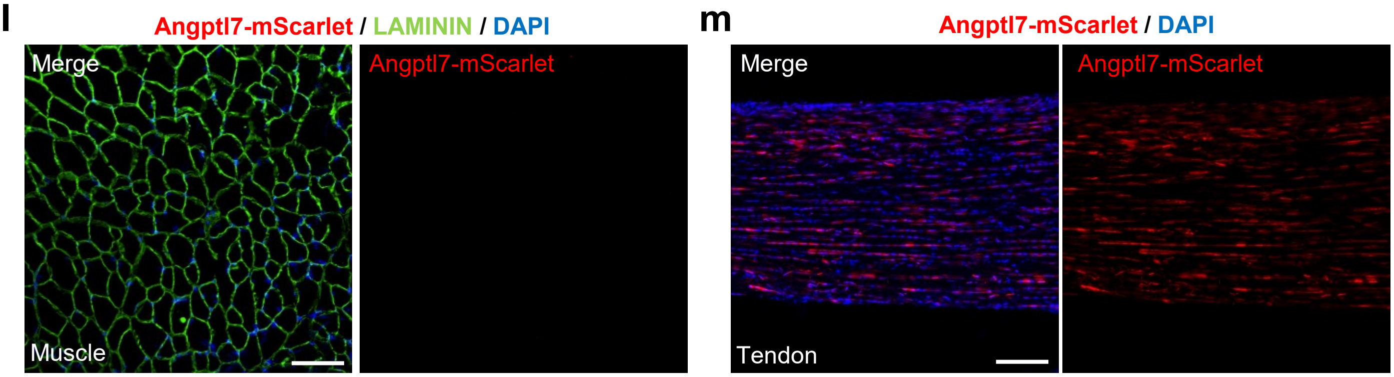

Supplementary information, Fig.S1. Identification of *Angptl7* as a specific marker for periosteal SSPC-1.

(a) Confocal imaging of femur sections from *Prrx1-Cre;Rosa26-Ai9* mice. Ct.B, cortical bone; PO, periosteum; BM, bone marrow. Scale bar: 100 µm.

(b) Representative Fluorescence activated cell sorting (FACS) plots of the Prrx1-Ai9$^+$ cells in the periosteum.

(c) Dotplots showing the marker genes for each population in the scRNA-seq of Prrx1-Ai9$^+$ cells.

(d-f) Bulk RNA-seq of P-SSCs and BM-SSCs. Schematic diagram of the experimental design (d). Representative FACS plots of the P-SSCs and BM-SSCs. SSCs: CD45$^-$CD31$^-$Ter119$^-$CD90$^-$6C3$^-$CD105$^-$CD200$^+$ (e). Heatmap of the top 15 DEGs highly expressed in P-SSCs than in BM-SSCs. Red, up-regulated; blue, down-regulated (f).

(g) Dotplots showing the marker genes for each population in the scRNA-seq of bone marrow Prrx1-lineage cells.

(h, i) UMAP plots visualizing the clustering of bone marrow Prrx1-lineage cells (h), and the expression of *Angptl7* in the scRNA-seq (i).

(j) UMAP plots visualizing the expression of *Angptl1*, *Angptl2*, *Angptl3*, *Angptl4* and *Angptl6* in the scRNA-seq from periosteal and bone marrow Prrx1-lineage cells.

(k) UMAP plots visualizing the expression of *Angpt1*, *Angpt2*, *Angpt4*, *Vegfa*, *Vegfb* and *Vegfc* in the scRNA-seq from periosteal and bone marrow Prrx1-lineage cells.

(l-m) Confocal imaging of muscle sections (l) and tendon sections (m) from 4-week-old Angptl7-mScarlet mice. Scale bar: 100 µm.
