# Supplementary material for: Fibrous-layer resident Angptl7+ periosteal stem cells sense injury inflammation to orchestrate fracture repair
Source: Cell Res. 2026 Jan 8;36(2):121–36. doi: 10.1038/s41422-025-01202-8 (PMC12847966; doi:10.1038/s41422-025-01202-8)
Supplement: Supplementary file 2 — Supplementary information, Fig.S2. Identification of Angptl7-lineage cells as P-SSCs in the fibrous-layer of periosteum [file 41422_2025_1202_MOESM2_ESM.pdf]

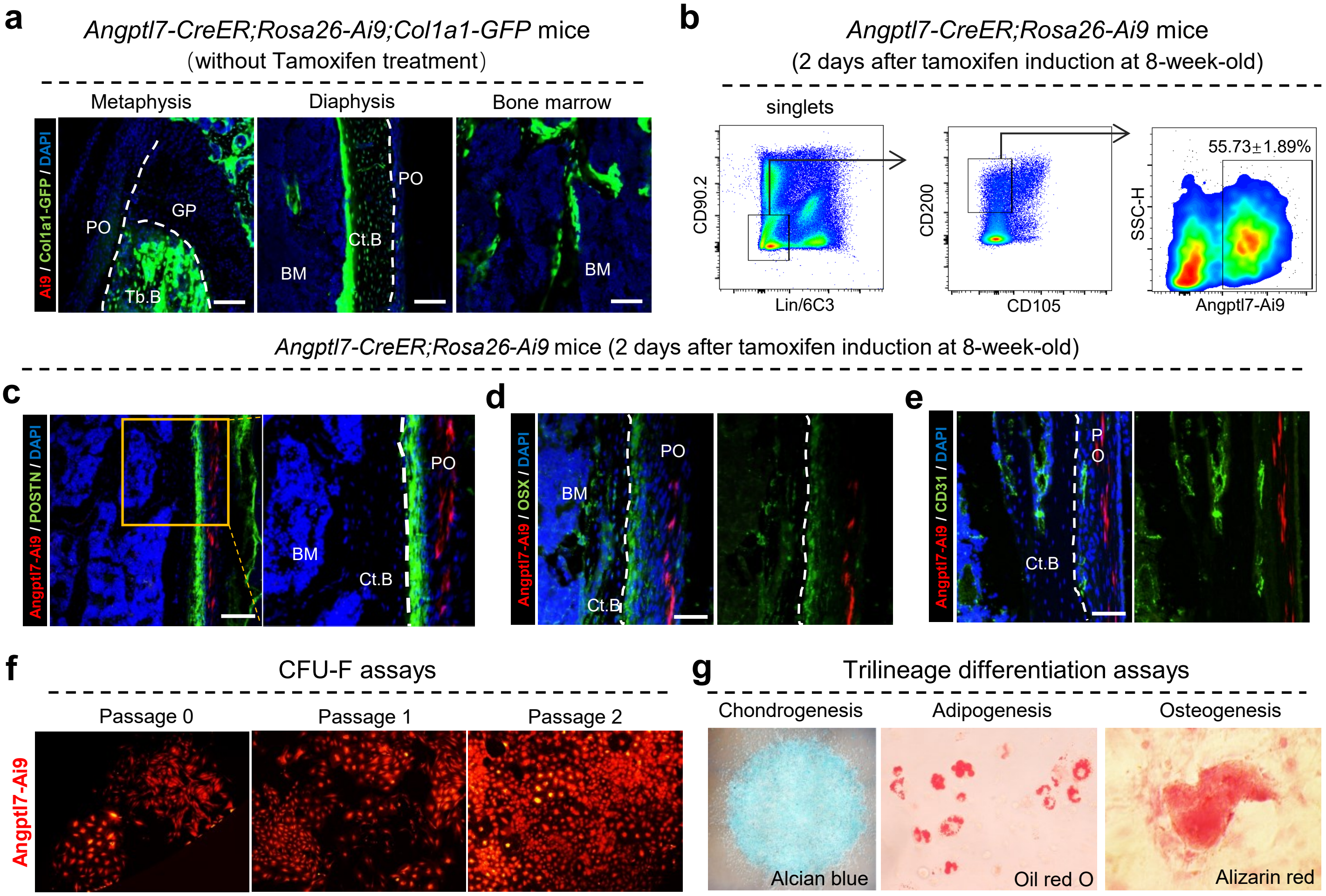

Supplementary information, Fig.S2. Identification of Angptl7-lineage cells as P-SSCs in the fibrous-layer of periosteum.

(a) Confocal imaging of femur sections from *Angptl7-CreER;Rosa26-Ai9;Col1a1-GFP* mice without tamoxifen induction. GP, growth plate. Scale bar: 100 μm.

(b) Flow cytometry showing the percentage of Angptl7-Ai9$^+$ in CD45$^-$CD31$^-$Ter119$^-$CD90$^-$6C3$^-$CD105$^-$CD200$^+$ SSCs in the periosteum from *Angptl7-CreER;Rosa26-Ai9* mice at 2 days after tamoxifen induction. Data represent mean ± SD. n=3 mice per condition.

(c-e) Confocal imaging of femur sections from *Angptl7-CreER;Rosa26-Ai9* mice showing the relative localization of Angptl7-lineage cells with POSTN (c), OSX (d) and CD31 (e). Mice were analyzed after 2 days when induced with tamoxifen at 8-week-old. Scale bar: 100 μm in (c) and 50 μm in (d, e).

(f) Colony forming unit-fibroblast assays of sorted Angptl7-Ai9$^+$ cells. Representative images showing the single-cell-driven colony formation of Angptl7-Ai9$^+$ cells.

(g) In vitro tri-lineage differentiation of sorted Angptl7-Ai9$^+$ cells. Representative images showing the alcian blue staining (left), Oil Red O staining (middle), and alizarin red staining (right) after 7 days culture of Angptl7-Ai9$^+$ cells with conditioned medium.
