# Supplementary material for: Fibrous-layer resident Angptl7+ periosteal stem cells sense injury inflammation to orchestrate fracture repair
Source: Cell Res. 2026 Jan 8;36(2):121–36. doi: 10.1038/s41422-025-01202-8 (PMC12847966; doi:10.1038/s41422-025-01202-8)
Supplement: Supplementary file 3 — Supplementary information, Fig.S3. Angptl7-lineage P-SSCs display minimal participation in postnatal bone development [file 41422_2025_1202_MOESM3_ESM.pdf]

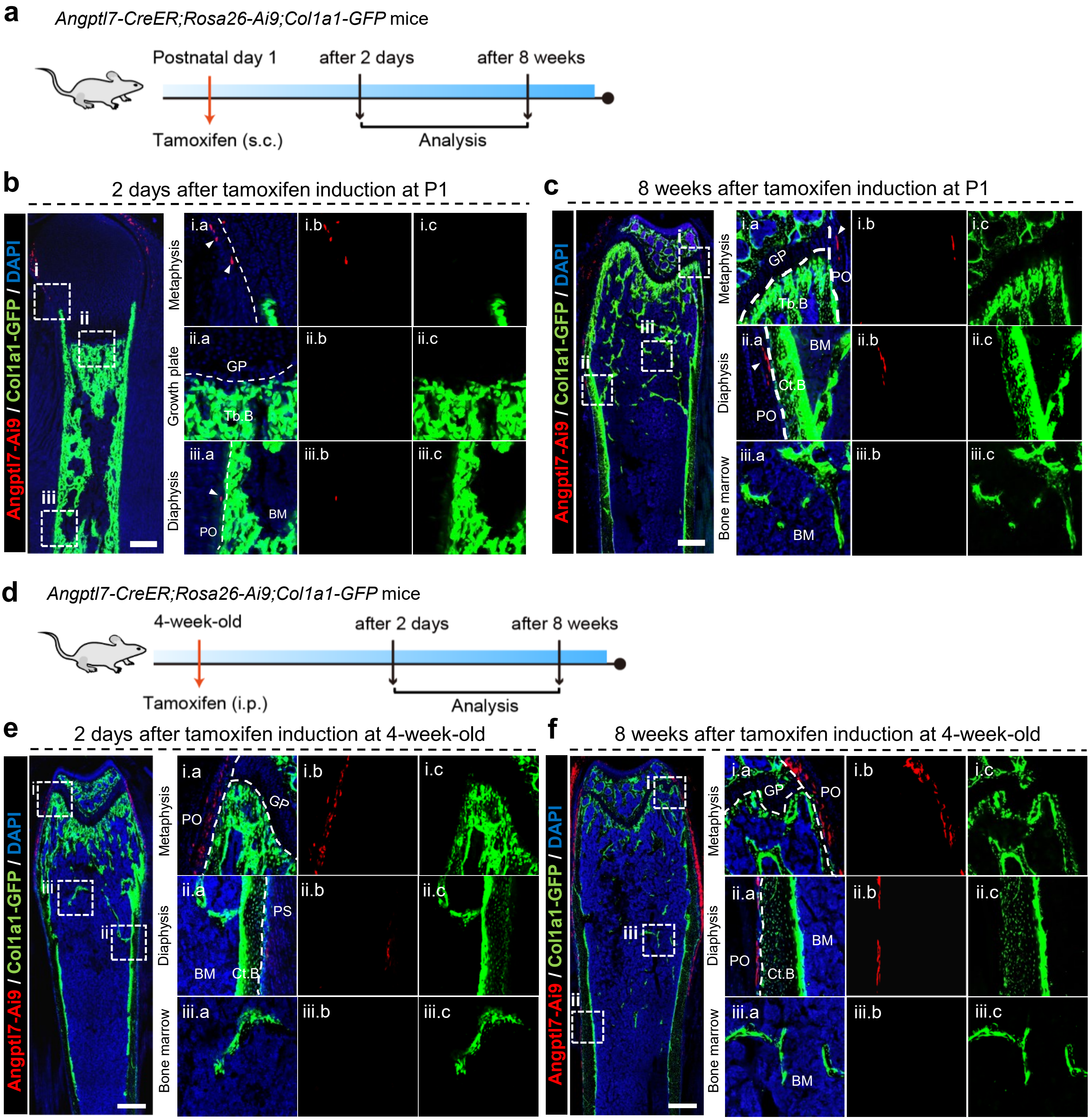

Supplementary information, Fig.S3. Angptl7-lineage P-SSCs display minimal participation in postnatal bone development.

(a) The diagram of tamoxifen induction strategy.

(b, c) Confocal imaging of femur sections from *Angptl7-CreER;Rosa26-Ai9;Col1a1-GFP* at 2 days (b) and 8 weeks (c) after tamoxifen treatment at P1. Scale bar: 250 μm in (b) and 500 μm in (c).

(d) The diagram of tamoxifen induction strategy.

(e, f) Confocal imaging of femur sections from *Angptl7-CreER;Rosa26-Ai9;Col1a1-GFP* mice that tamoxifen induced at 4-week-old. Mice were analyzed at 2 day (e) and 8 weeks (f) after induction. Scale bar: 500 μm.
