# Supplementary material for: Fibrous-layer resident Angptl7+ periosteal stem cells sense injury inflammation to orchestrate fracture repair
Source: Cell Res. 2026 Jan 8;36(2):121–36. doi: 10.1038/s41422-025-01202-8 (PMC12847966; doi:10.1038/s41422-025-01202-8)
Supplement: Supplementary file 4 — Supplementary information, Fig.S4. Angptl7-lineage P-SSCs are basically distinguished from CD34-lineage fibroblasts [file 41422_2025_1202_MOESM4_ESM.pdf]

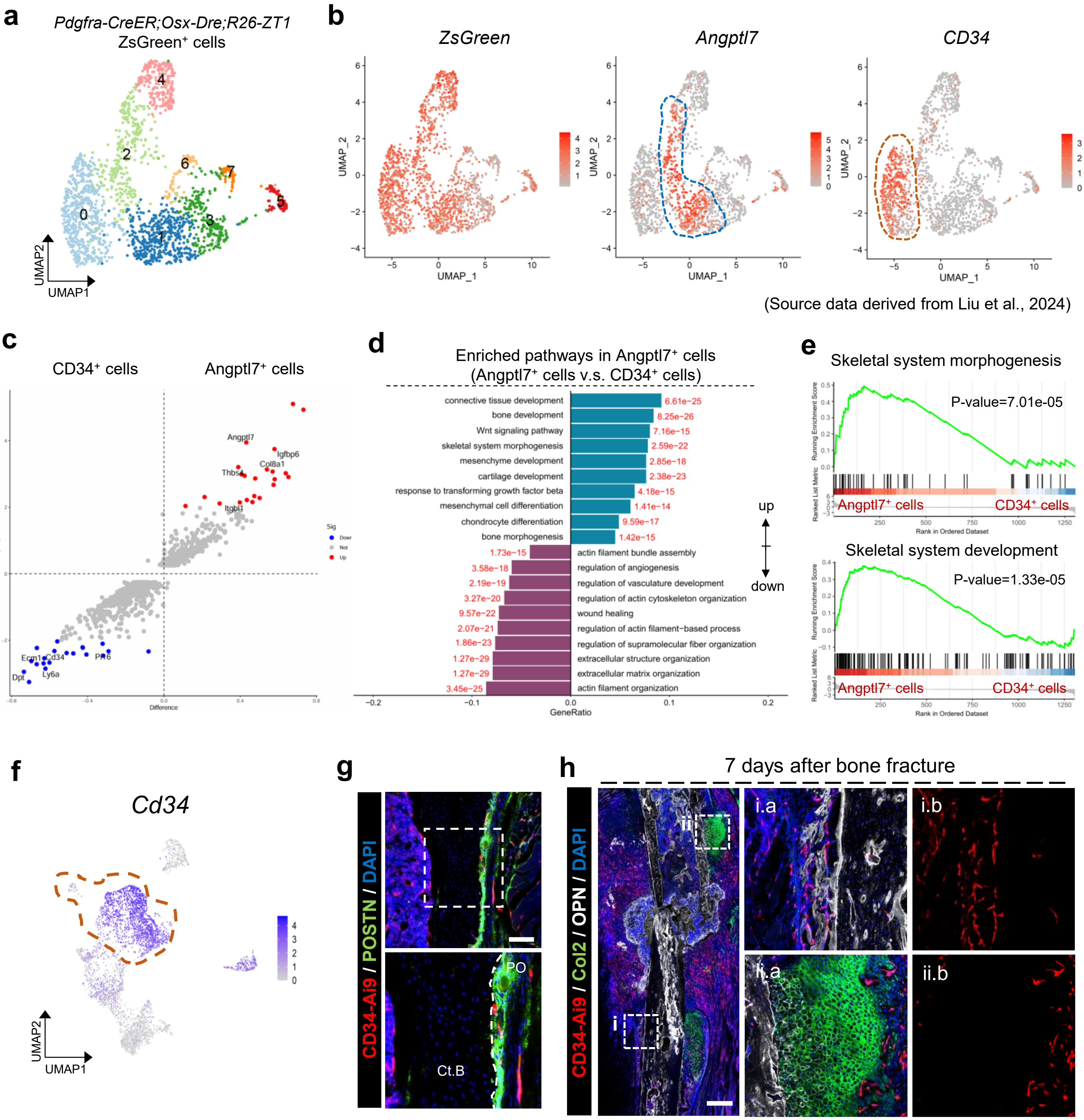

Supplementary information, Fig.S4. Angptl7-lineage P-SSCs are basically distinguished from CD34-lineage fibroblasts.

(a, b) UMAP plots showing the re-clustering results (a), and the expression of *ZsGreen*, *Angptl7* and *CD34* (b) in the scRNA-seq data of ZsGreen⁺ fibrous-layer periosteal cells from *Pdgfra-CreER;Osx-Dre;ZT1* mice.

(c) Mean-Average plots showing the DEGs between Angptl7⁺ cells and CD34⁺ cells. Red, high expressed in Angptl7⁺ cells; blue, high expressed in CD34⁺ cells.

(d) GO analysis of the DEGs between Angptl7⁺ cells and CD34⁺ cells.

(e) GSEA analysis showing the enriched gene signature in Angptl7⁺ cells.

(f) UMAP plots of *CD34* in the scRNA-seq data of periosteal Prrx1-Ai9⁺ cells.

(g) Confocal imaging of femur sections showing the CD34-lineage cells and their relative localization with POSTN at 2 days after tamoxifen treatment at 8-week-old. Scale bar: 100 μm.

(h) Confocal imaging of femur sections from *CD34-CreER;Rosa26-Ai9* mice at 7 days after fracture. High-magnification views showing on the right. Scale bar: 500 μm.
