# Supplementary material for: Fibrous-layer resident Angptl7+ periosteal stem cells sense injury inflammation to orchestrate fracture repair
Source: Cell Res. 2026 Jan 8;36(2):121–36. doi: 10.1038/s41422-025-01202-8 (PMC12847966; doi:10.1038/s41422-025-01202-8)
Supplement: Supplementary file 6 — Supplementary information, Fig.S6. ScRNA-seq analysis of the Angptl7-lineage cells during fracture repair [file 41422_2025_1202_MOESM6_ESM.pdf]

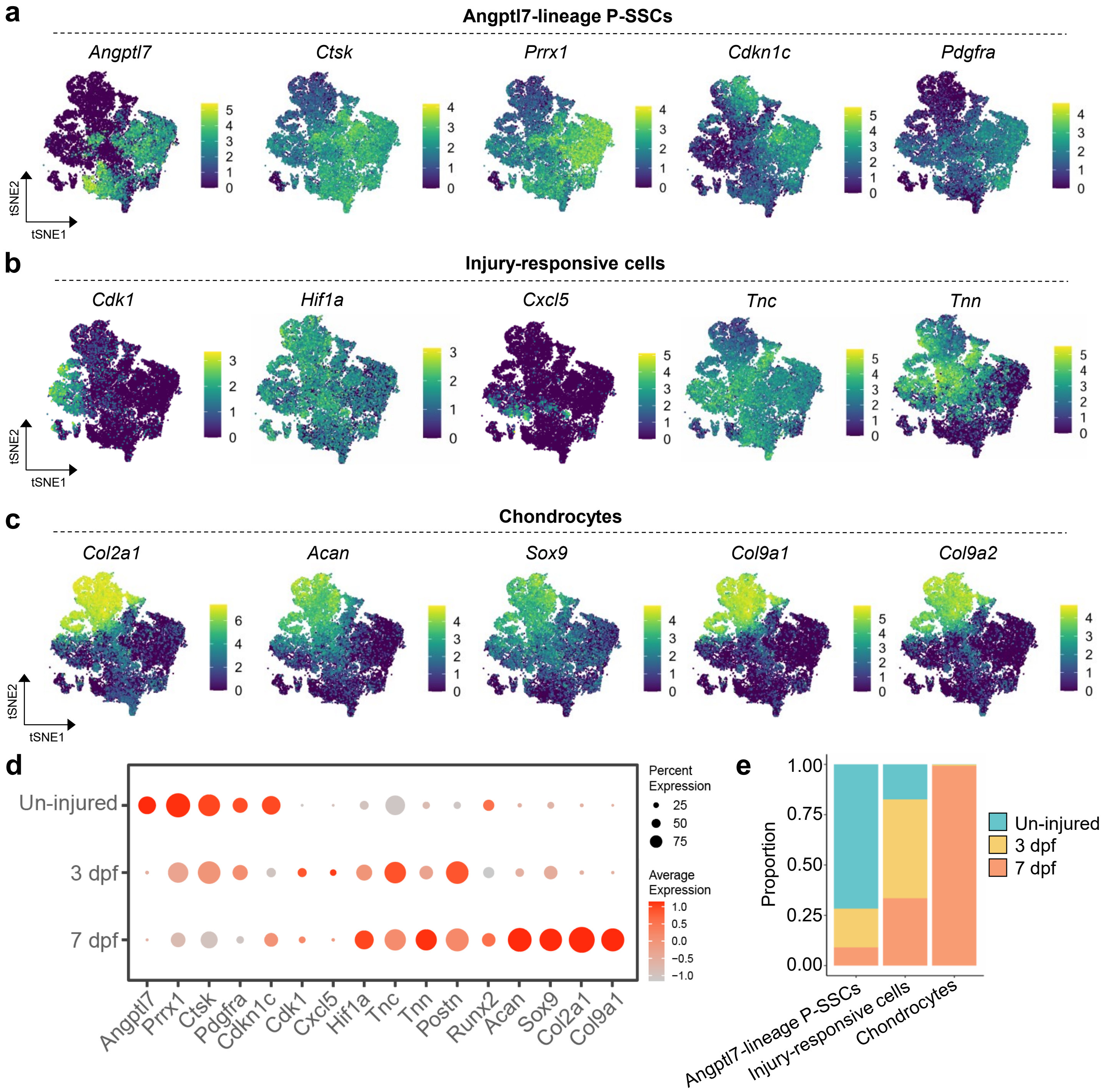

Supplementary information, Fig.S6. ScRNA-seq analysis of the Angptl7-lineage cells during fracture repair.

(a) tSNE plots showing the expression of *Angptl7, Ctsk, Prrx1, Cdkn1c* and *Pdgfra* in the scRNA-seq.

(b) tSNE plots showing the expression of *Cdk1, Hif1a, Cxcl5, Tnc* and *Tnn* in the scRNA-seq.

(c) tSNE plots showing the expression of *Col2a1, Acan, Sox9, Col9a1* and *Col9a2* in the scRNA-seq.

(d) Dotplots showing the signature genes in the scRNA-seq grouped by samples from uninjured femurs, fractured femurs at 3 dpf, and callus from 7 dpf.

(e) Barplots showing relative contribution of uninjured, 3 dpf and 7 dpf samples across clusters in the scRNA-seq.
