# Supplementary material for: Fibrous-layer resident Angptl7+ periosteal stem cells sense injury inflammation to orchestrate fracture repair
Source: Cell Res. 2026 Jan 8;36(2):121–36. doi: 10.1038/s41422-025-01202-8 (PMC12847966; doi:10.1038/s41422-025-01202-8)
Supplement: Supplementary file 7 — Supplementary information, Fig.S7. Dynamic expression of angiogenic factors in Angptl7-lineage cells during fracture repair [file 41422_2025_1202_MOESM7_ESM.pdf]

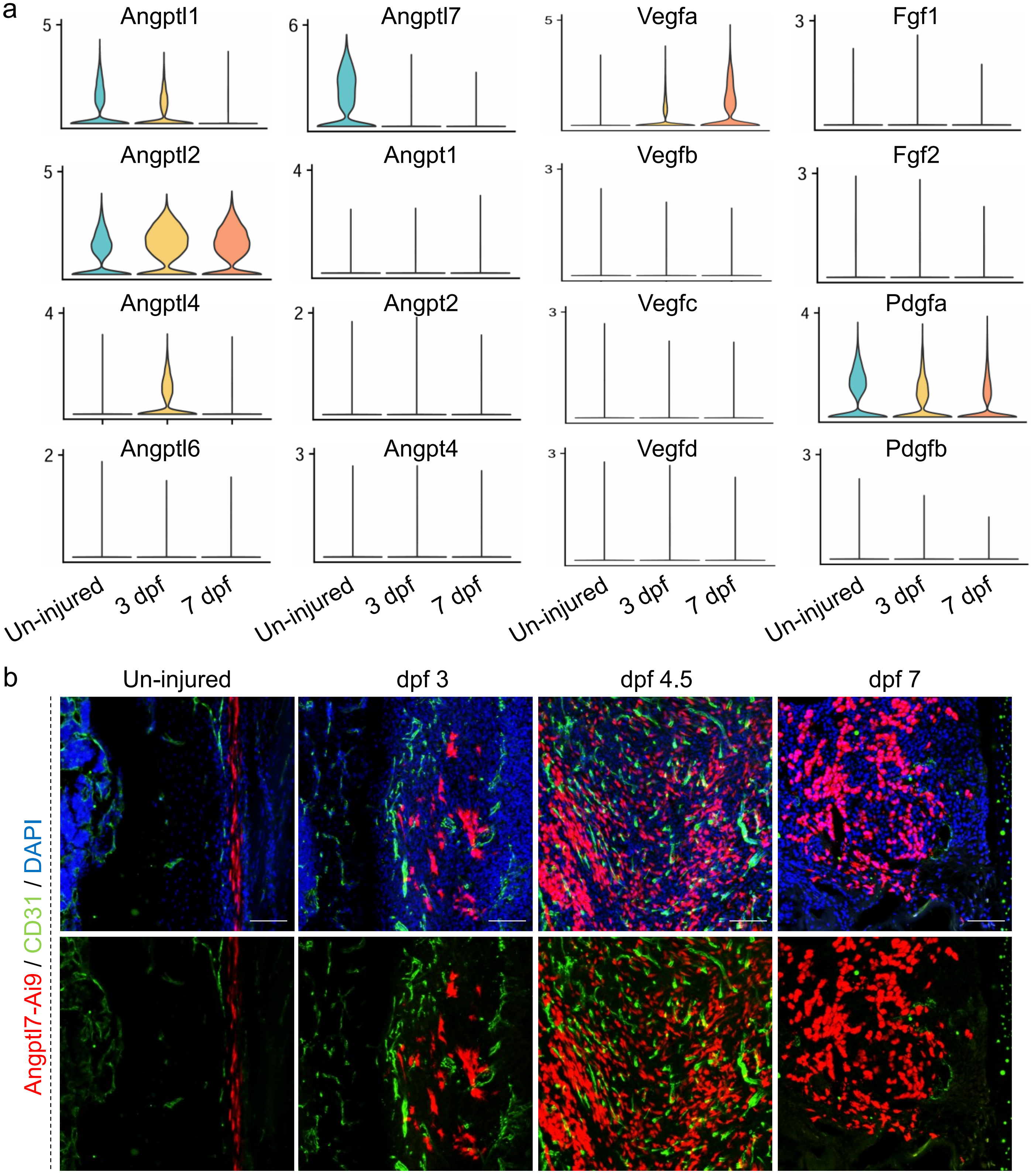

Supplementary information, Fig.S7. Dynamic expression of angiogenic factors in Angptl7-lineage cells during fracture repair.

(a) Violin plots showing the dynamic expression of various angiogenic factors in Angptl7-lineage cells during fracture.

(b) Fluorescence imaging showing the dynamic spatial relationships between Angptl7-lineage cells and $CD31^{+}$ endothelial cells during bone fracture. Scale bar: 100 μm.
