# Supplementary material for: Fibrous-layer resident Angptl7+ periosteal stem cells sense injury inflammation to orchestrate fracture repair
Source: Cell Res. 2026 Jan 8;36(2):121–36. doi: 10.1038/s41422-025-01202-8 (PMC12847966; doi:10.1038/s41422-025-01202-8)
Supplement: Supplementary file 5 — Supplementary information, Fig.S5. Functional perturbation of Angptl7-lineage P-SSCs exhibits negligible effects on bone homeostasis [file 41422_2025_1202_MOESM5_ESM.pdf]

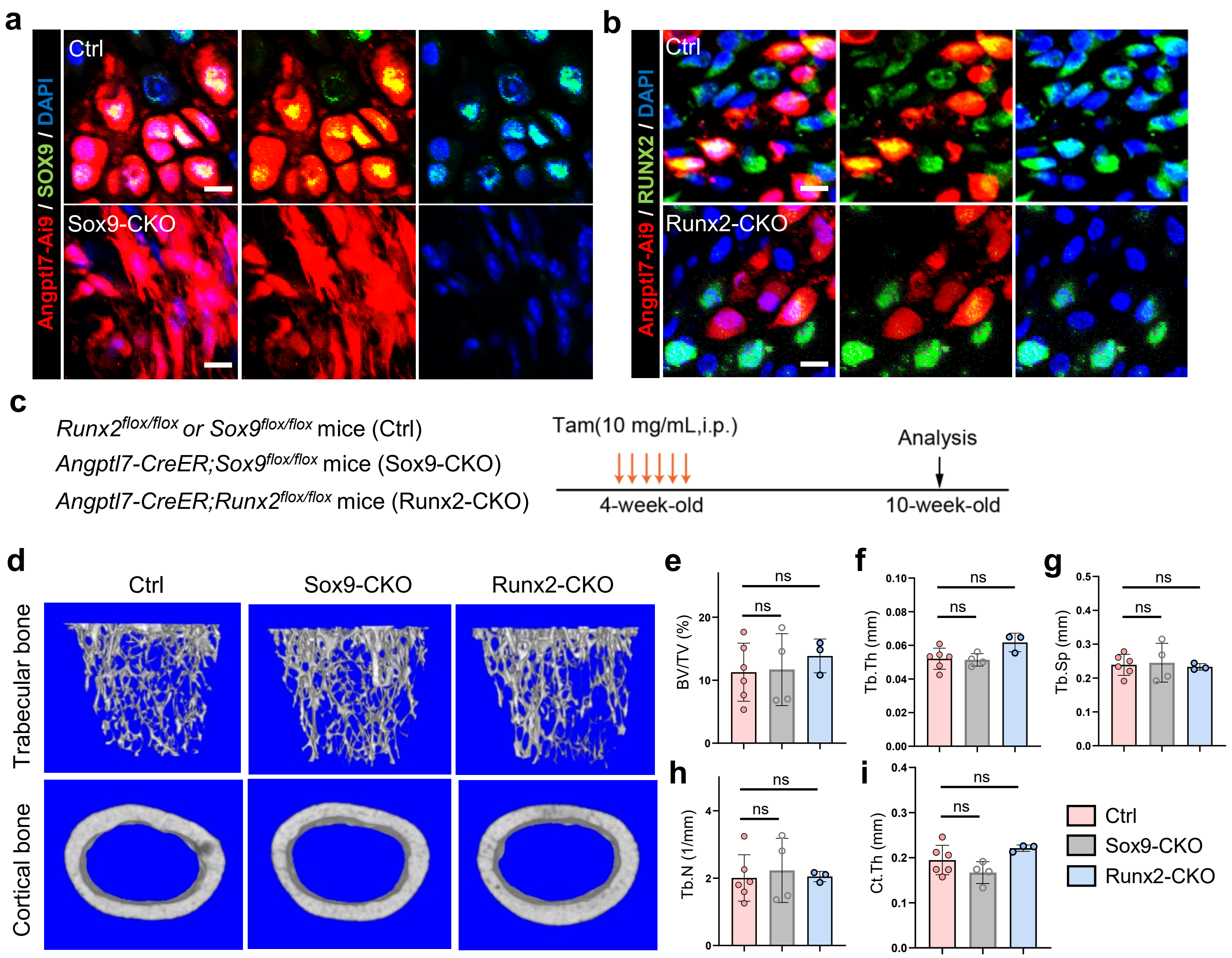

Supplementary information, Fig.S5. Functional perturbation of Angptl7-lineage P-SSCs exhibits negligible effects on bone homeostasis.

(a) Immunostaining of SOX9 in *Angptl7-CreER;Rosa26-Ai9* (Ctrl) and *Angptl7-CreER;Sox9$^{fl/fl}$;Rosa26-Ai9* (Sox9-CKO) mice at 7 dpf. Scale bar: 10 μm.

(b) Immunostaining of Runx2 in *Angptl7-CreER;Rosa26-Ai9* (Ctrl) and *Angptl7-CreER;Runx2$^{fl/fl}$;Rosa26-Ai9* mice (Runx2-CKO) at 14 dpf. Scale bar: 10 μm.

(c) The diagram of tamoxifen induction strategy.

(d) Representative images showing the μCT re-construction of trabecular bone and cortical bone of the femurs from Runx2-CKO, Sox9-CKO and control mice.

(e-i) Quantification of bone density of the femurs from Runx2-CKO, Sox9-CKO and control mice. Data represent mean ± SD. two-tailed unpaired t-test. n≥3 mice per condition.
